# Selective Binding of Small Molecules to *Vibrio cholerae* DsbA Offers a Starting Point for the Design of Novel Antibacterials

**DOI:** 10.1002/cmdc.202100673

**Published:** 2022-01-27

**Authors:** Geqing Wang, Biswaranjan Mohanty, Martin L. Williams, Bradley C. Doak, Rabeb Dhouib, Makrina Totsika, Róisín M. McMahon, Gaurav Sharma, Dan Zheng, Matthew R. Bentley, Yanni Ka‐Yan Chin, James Horne, David K. Chalmers, Begoña Heras, Martin J. Scanlon

**Affiliations:** ^1^ Department of Biochemistry and Genetics La Trobe Institute for Molecular Science La Trobe University 3083 Melbourne Victoria Australia; ^2^ Medicinal Chemistry Monash Institute of Pharmaceutical Sciences Monash University 381 Royal Parade 3052 Parkville Victoria Australia; ^3^ Queensland University of Technology Institute of Health and Biomedical Innovation School of Biomedical Sciences Brisbane Queensland Australia; ^4^ Griffith Institute for Drug Discovery Griffith University 4111 Nathan Queensland Australia; ^5^ Institute for Molecular Bioscience The University of Queensland 4072 St Lucia Queensland Australia; ^6^ Centre for Advanced Imaging The University of Queensland 4072 St Lucia Queensland Australia; ^7^ Central Science Laboratory The University of Tasmania 7005 Sandy Bay Tasmania Australia; ^8^ ARC Training Centre for Fragment Based Design Monash Institute of Pharmaceutical Sciences Monash University 381 Royal Parade 3052 Parkville Victoria Australia; ^9^ Current Address: Sydney Analytical Core Research Facility The University of Sydney 2006 Sydney New South Wales Australia

**Keywords:** oxidoreductase, DsbA, fragment-based drug discovery, bile salt, Vibrio cholerae, antibacterials

## Abstract

DsbA enzymes catalyze oxidative folding of proteins that are secreted into the periplasm of Gram‐negative bacteria, and they are indispensable for the virulence of human pathogens such as *Vibrio cholerae* and *Escherichia coli*. Therefore, targeting DsbA represents an attractive approach to control bacterial virulence. X‐ray crystal structures reveal that DsbA enzymes share a similar fold, however, the hydrophobic groove adjacent to the active site, which is implicated in substrate binding, is shorter and flatter in the structure of *V. cholerae* DsbA (VcDsbA) compared to *E. coli* DsbA (EcDsbA). The flat and largely featureless nature of this hydrophobic groove is challenging for the development of small molecule inhibitors. Using fragment‐based screening approaches, we have identified a novel small molecule, based on the benzimidazole scaffold, that binds to the hydrophobic groove of oxidized VcDsbA with a *K*_D_ of 446±10 μM. The same benzimidazole compound has ∼8‐fold selectivity for VcDsbA over EcDsbA and binds to oxidized EcDsbA, with *K*_D_>3.5 mM. We generated a model of the benzimidazole complex with VcDsbA using NMR data but were unable to determine the structure of the benzimidazole bound EcDsbA using either NMR or X‐ray crystallography. Therefore, a structural basis for the observed selectivity is unclear. To better understand ligand binding to these two enzymes we crystallized each of them in complex with a known ligand, the bile salt sodium taurocholate. The crystal structures show that taurocholate adopts different binding poses in complex with VcDsbA and EcDsbA, and reveal the protein‐ligand interactions that stabilize the different modes of binding. This work highlights the capacity of fragment‐based drug discovery to identify inhibitors of challenging protein targets. In addition, it provides a starting point for development of more potent and specific VcDsbA inhibitors that act through a novel anti‐virulence mechanism.

## Graphical abstract

**DsbA is a key mediator** of bacterial virulence and an attractive antibacterial target. Fragment‐based screening approach has identified novel compounds that bind to the active site groove of DsbA from *Vibrio cholerae*. Biophysical characterisation of the fragment binder and bile salt sodium taurocholate uncovers the molecular basis of binding selectivity and provides a starting point for development of specific VcDsbA inhibitors.

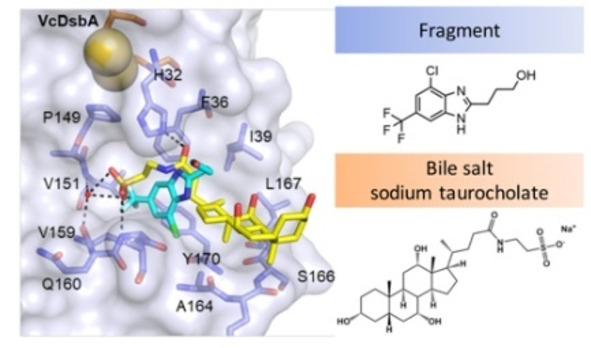

## Introduction

*Vibrio cholerae* is a Gram‐negative bacterium that causes cholera in humans. Cholera is an acute diarrhoeal disease with an estimated burden of 1.3 to 4.0 million cases and 21,000 to 143,000 deaths per year worldwide.^[1]^ Infection with *V. cholerae* is most commonly acquired through the ingestion of contaminated food or water.^[2]^ The virulence of *V. cholerae* is attributable to its deployment of multiple virulence factors, including cholera toxin (CT) and the toxin‐coregulated pilus (TCP).^[2b]^ CT acts on enterocytes, causing efflux of ions and water from the cells, leading to watery diarrhoea. TCPs promote host colonization by adhering to host microvilli and are pivotal to the pathogenesis of *V. cholerae*.^[3]^ The conformational maturation and secretion of virulence factors, including CT and TCP, depend on oxidative folding in the periplasm, which is catalyzed by the thiol‐disulfide oxidoreductase enzyme, *V. cholerae* DsbA (VcDsbA), encoded by the *tcpg* gene.^[4]^ Disruption of VcDsbA leads to defects in the functional folding and secretion of *V. cholerae* virulence factors. This, in turn has multiple phenotypic effects in *V. cholerae* including compromised colonization and auto‐agglutination.[^4^, ^5^] Therefore, inhibitors of VcDsbA may attenuate the virulence of *V. cholerae*.

DsbA enzymes from other Gram‐negative bacteria have been identified as key mediators of bacterial virulence and may therefore represent promising antibacterial drug targets. In many cases it has been shown that bacteria lacking a functional DsbA enzyme have reduced virulence, enhanced sensitivity to antibiotics and decreased capacity to initiate infection.^[6]^ For example, deletion of *dsbA* in uropathogenic *E. coli* (UPEC) strongly attenuated its ability to colonize the bladder^[7]^ and *dsbA* mutants rendered *Salmonella enterica* serovar Typhimurium avirulent.^[8]^ DsbA enzymes catalyze disulfide bond formation in the periplasm of Gram‐negative bacteria. The loss of virulence in *dsbA* mutants can often be linked to the misfolding of a DsbA substrate.^[9]^

**Figure 1:**
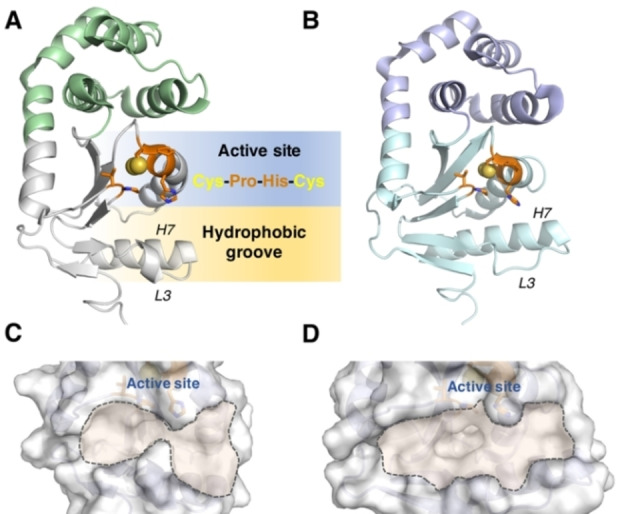
Crystal structures of VcDsbA and EcDsbA. **A**. Crystal structure of oxidized VcDsbA (PDB code: 4DVC). The thioredoxin domain is coloured in white, α‐helical domain is coloured in green. α‐helix H7 and loop L3, which form a part of the hydrophobic groove are labelled in the structure. Active site residues V148, *cis*‐P149, C30, P31, H32 and C33 are shown as orange sticks. Sulfur atoms of redox active cysteines are shown as spheres. **B**. Crystal structure of oxidized EcDsbA (PDB code: 1FVK). The thioredoxin domain is coloured in cyan, α‐helical domain is coloured in purple. α‐helix H7 and loop L3, which form a part of the hydrophobic groove, are labelled in the structure. Active site residues V150, *cis*‐P151, C30, P31, H32 and C33 are shown as orange sticks. Sulfur atoms of redox active cysteines are shown as spheres. **C.–D**. The hydrophobic grooves of VcDsbA and EcDsbA are highlighted by dashed lines on the surface models. The active sites are labelled in the structures.

DsbA enzymes retain a conserved tertiary structure, comprising a classical thioredoxin (TRX) domain with an inserted α‐helical domain (Figure 1A–B). The TRX domain contains a pair of redox‐active cysteine residues in a CXXC motif located adjacent to a conserved *cis*‐proline residue (Figure 1A).^[10]^ These features contribute to a hydrophobic patch of residues located in a surface groove of DsbA that is required for substrate recognition.^[11]^ In the oxidative folding reaction, cysteine residues at the active site of DsbA cycle between their oxidized (disulfide) and reduced (dithiol) state. Oxidized DsbA reacts with reduced protein substrates to form a mixed disulfide intermediate.^[12]^ This covalent intermediate is resolved to release the oxidized substrate and reduced DsbA. To prime for the next redox cycle, the active site cysteine pair of reduced DsbA is re‐oxidized by the inner membrane protein DsbB in concert with its cofactor ubiquinone, restoring DsbA to its active oxidized state.^[13]^

The hydrophobic groove of DsbA enzymes mediates interaction with both DsbB and substrates. The relatively flat and featureless surface of the groove represents a significant obstacle for inhibitor design (Figure 1C–D).^[14]^ Nonetheless, small molecule inhibitors targeting DsbA enzymes have been identified.^[15]^ Covalent peptide inhibitors of *E. coli* DsbA (EcDsbA) have been developed based on the sequence of the loop in EcDsbB that interacts directly with EcDsbA.^[16]^ Additionally, small molecule inhibitors of EcDsbA have been developed using fragment‐based screening methods.[^15b^, ^15f^, ^16^]

Although VcDsbA shares the canonical DsbA fold, there are some differences between the active sites of VcDsbA and EcDsbA (Figure 1). In particular VcDsbA possesses a more truncated and flatter groove compared to EcDsbA due to a three‐residue deletion in loop L3 and a four‐residue deletion in helix H7.^[17]^ In this work, we sought to identify small molecule inhibitors of VcDsbA using a fragment‐based screening strategy and to characterize their binding to VcDsbA. In addition, to examine difference in ligand recognition between these two enzymes, structures of their complexes with a known ligand, the bile salt sodium taurocholate (TC),^[18]^ were characterized by X‐ray crystallography. Together these structural and binding data highlight important differences between EcDsbA and VcDsbA in the context of inhibitor design. In addition, the compounds described here provide starting points for the future development of more potent VcDsbA inhibitors.

## Results

### Fragment‐based discovery of VcDsbA inhibitors

**Figure 2:**
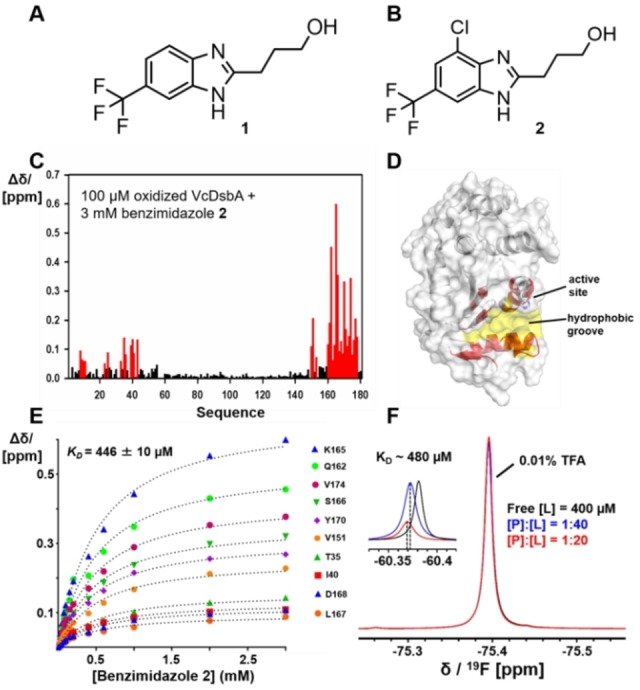
Characterization of benzimidazole 2 binding to VcDsbA. **A**. Chemical structure of the initial hit benzimidazole **1. B**. Chemical structure of the best benzimidazole **2. C**. Histogram showing chemical shift perturbations (CSP) observed in the ^1^H‐^15^N HSQC spectrum of VcDsbA (100 μM) upon addition of 3 mM benzimidazole **2**. Residues with CSP greater than 0.05 ppm are shown in red **D**. Residues with large CSP are coloured red and mapped onto the crystal structure of VcDsbA. The protein surface formed by hydrophobic groove residues is shown in yellow. **E**. CSP were measured with increasing concentrations of benzimidazole **2** to estimate the binding affinity (*K*_D_). **F**. 1D ^19^F‐NMR spectra of benzimidazole **2** at different concentration ratios of unlabelled oxidized VcDsbA and benzimidazole **2**. VcDsbA concentration was held constant at 10 μM and the benzimidazole **2** concentration was varied as indicated. Spectra were referenced and normalized to the resonance of an internal standard of trifluoroacetic acid (0.01 % *v/v*).

To identify small molecules that bound to oxidized VcDsbA, a library of 500 fragments from Maybridge (ThermoFisher Scientific) was screened by 1D ^1^H Saturation Transfer Difference (STD) NMR spectroscopy. Fifteen fragments were identified as hits (Figure S1) based on the intensity of signals observed in their STD spectra^[19]^ and their binding was confirmed by recording ^1^H‐^15^N HSQC spectra of VcDsbA in the absence and presence of each fragment. The trifluoromethyl‐substituted benzimidazole **1** (Figure 2), was observed to elicit the largest chemical shift perturbations (CSP) of peaks in the HSQC spectrum for residues located in the hydrophobic groove of VcDsbA (Figure S2A). Therefore, this fragment was selected for further investigation. The binding affinity was estimated by recording a series of ^1^H‐^15^N HSQC spectra in the presence of increasing concentrations of benzimidazole **1**, which yielded an equilibrium dissociation constant (*K*_D_) of 610±150 μM. To generate preliminary structure‐binding relationships, seven benzimidazole analogues were tested for their ability to bind to VcDsbA as listed in Supporting Information Figure S2. The analogues were designed to evaluate which portions of benzimidazole **1** were essential for binding, and to identify positions that could be developed to improve affinity. The binding of these analogues was evaluated by monitoring the extent of CSP observed in ^1^H‐^15^N HSQC upon addition of the compounds (1 mM). Two of the seven analogues elicited larger CSP in ^1^H‐^15^N HSQC of VcDsbA than the parent benzimidazole, suggesting that they bound with higher affinity (Figure S2). Benzimidazole **2** (Figure 2B) gave the largest CSP in the HSQC spectra and was selected for additional characterization.

To map the binding site, ^15^N‐labeled oxidized VcDsbA (100 μM) was titrated with benzimidazole **2** (0.05 mM to 3 mM) at 298 K. The CSP observed in the ^1^H‐^15^N HSQC spectra of VcDsbA showed linear perturbations with increasing ligand concentration, consistent with 1 : 1 binding stoichiometry and indicating that the complex was in fast exchange on the NMR chemical shift time scale. Residues with a CSP >0.05 ppm at the highest ligand concentration tested were mapped onto the VcDsbA structure (Figure 2C, D). The residues showing the largest CSP were localized within the hydrophobic groove, adjacent to the active site (Figure 2D). The CSP titration data were fitted to a one‐site binding model, which yielded a *K*_D_ of 446±10 μM for the VcDsbA – benzimidazole **2** interaction (Figure 2E). The *K*_D_ for the VcDsbA‐benzimidazole **2** interaction was also estimated using ligand‐detected 1D ^19^F‐NMR (Figure 2F). The ^19^F chemical shift of the CF_3_ group in benzimidazole **2** has a chemical shift of −60.38 ppm in the absence of protein and was perturbed upon in a concentration‐dependent fashion upon addition of VcDsbA. The change in the chemical shift of the ^19^F signal at two different protein : ligand ratios was used to estimate the *K*_D_ of the interaction.^[20]^ This analysis produced a value of *K*_D_=480 μM (Figure 2F), which is in good agreement with the value calculated from the ^1^H‐^15^N HSQC titrations.

Since benzimidazole **2** binds into the hydrophobic groove of VcDsbA, we also evaluated its ability to bind to the corresponding hydrophobic groove in EcDsbA, which is larger than that of VcDsbA. HSQC titration showed that benzimidazole **2** binds with low affinity to the hydrophobic groove of oxidized EcDsbA. The titration did not reach saturation at the highest concentration of benzimidazole **2** that was tested, and analysis of the data revealed that it bound with a *K*_D_ of >3.5 mM (Figure S3), at least 8‐fold weaker than its affinity to VcDsbA. The pattern of CSP in the HSQC spectra of EcDsbA upon titration with benzimidazole **2** was slightly different to that observed for VcDsbA. In addition to the CSP observed for residues in the hydrophobic groove, a cluster of residues on the opposite face of EcDsbA were also perturbed, suggesting benzimidazole **2** may bind both to the hydrophobic groove and in the cleft between the two domains of EcDsbA. Small molecules have previously been identified that bind in a similar location on *Pseudomonas aeruginosa* DsbA and *Burkholderia pseudomallei* DsbA.[^15g^, ^19^]

### Characterization of benzimidazole 2 binding mode with VcDsbA

**Figure 3:**
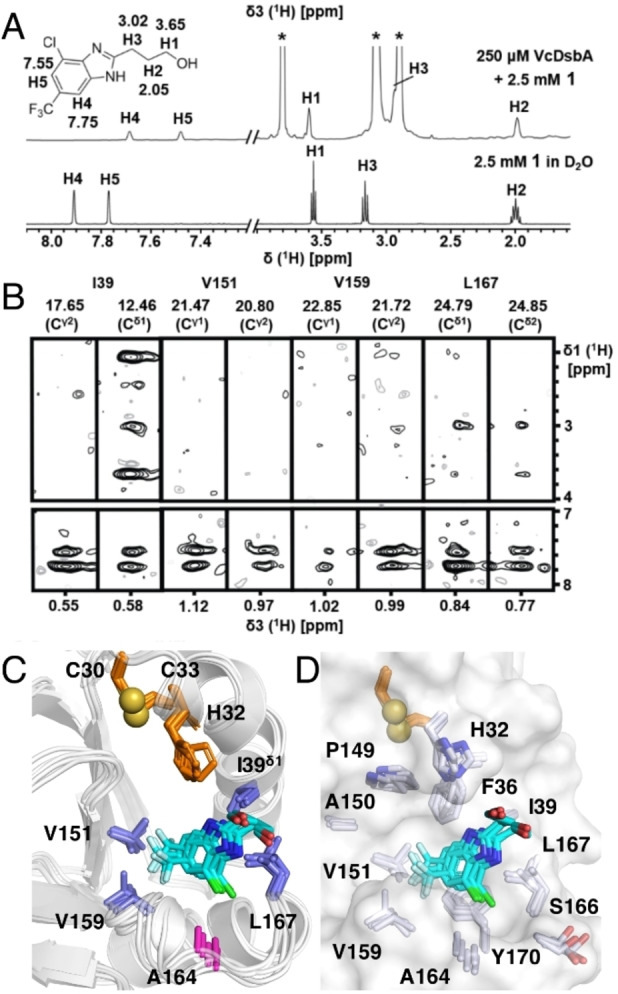
HADDOCK docking model of oxidized VcDsbA in complex with benzimidazole 2. **A**. Structure of benzimidazole **2** with ^1^H NMR assignments in the presence (top) and absence (bottom) of oxidized VcDsbA. * indicates buffer signals. **B**. 2D [^1^H,^1^H]‐NOESY strip plot of 3D ω1‐^13^C,^15^N‐filtered, ω3‐^13^C_ali_(methyl) edited [^1^H,^1^H]‐NOESY spectrum of oxidized VcDsbA in the presence of benzimidazole **2**. Strips are shown for residues where intermolecular NOEs with benzimidazole **2** were observed. **C**. Five lowest‐energy HADDOCK models of the VcDsbA–benzimidazole **2** complex. The protein is shown as a cartoon and the ligand in stick representation. Residues for which intermolecular NOEs with benzimidazole **2** were observed are shown in blue sticks and labelled. No intermolecular NOEs were observed from benzimidazole **2** to A164‐CH_3_, this residue is located at the lower edge of hydrophobic groove of VcDsbA. The side chain of A164 is shown in magenta. **D**. Surface representation of VcDsbA in the same orientation as (C) with residues within 5 Å of benzimidazole **2** shown as sticks. In both panels C and D active site side chain residues are shown as orange sticks and the sulfur atoms of C30 and C33 as yellow spheres.

To understand the selectivity of benzimidazole **2** binding, we next sought to generate structures in complex with VcDsbA and EcDsbA. Attempts to soak and co‐crystallize benzimidazole **2** with VcDsbA and EcDsbA were unsuccessful, and no electron density of the compound was observed in the resulting crystal structures. Therefore we determined an NMR model of the complex with VcDsbA using a previously described protocol.^[21]^ The solubility of benzimidazole **2** in aqueous NMR buffer was established by recording a 2D [^1^H,^1^H]‐NOESY spectrum (NOE mixing time of 800 ms) which revealed that NOE cross peaks were the opposite phase to the diagonal. This observation suggests that benzimidazole **2** was soluble and free from aggregation at the concentration of 2.5 mM used in this experiment (Figure S4). A 3D ω_1_‐^13^C,^15^N‐filtered, ω_3_‐^13^C_ali_(methyl) edited [^1^H,^1^H]‐NOESY was acquired using a small spectral width selecting only the methyl region in the indirect carbon dimension,^[22]^ on a sample containing uniformly ^13^C,^15^N‐labeled VcDsbA (0.25 mM) and unlabeled benzimidazole **2** (2.5 mM) with 2 % (*v/v*) d_6_‐DMSO. We observed a total of 21 intermolecular NOEs between oxidized VcDsbA and benzimidazole **2** (Table S2). NOEs were observed to the methyl resonances of I39, V151, V159 and L167 of VcDsbA (Figure 3B). No intermolecular NOEs were detected to A164, which is the only methyl‐containing residue in the dynamic loop L3 of the VcDsbA hydrophobic groove. Using the intermolecular NOEs and ^1^H‐^15^N HSQC CSP as ambiguous interaction restraints (AIR), we generated models of the VcDsbA‐benzimidazole **2** complex using HADDOCK,^[23]^ using a protocol described previously.^[21]^ A final set of 10 conformers was selected to represent the structure of the complex, which had the lowest HADDOCK scores and restraint violation energies. In all models, benzimidazole **2** was found in a similar orientation. The largest cluster comprising 5 out of 10 conformers is shown in Figure 3C–D. In this NMR model, the benzimidazole **2** core and chlorine group make hydrophobic and π‐π interactions to F36, I39, L167, and Y170 while the CF_3_ group makes hydrophobic contacts with V151 and V159.

### Characterizing the binding site and binding interactions of TC to oxidized VcDsbA and EcDsbA by NMR spectroscopy

Xue *et al*. have reported that TC binds to VcDsbA and EcDsbA with *K*_D_ values of 40±2.5 μM and 146±7 μM, respectively, as measured by ITC.^[18]^ The same study reported that TC was able to affect the redox state of VcDsbA and inhibit VcDsbA substrate oxidation *in vivo*,^[18]^ suggesting TC may bind to the hydrophobic groove. To characterize the binding site and binding modes of TC, we characterized the interaction of TC with EcDsbA and VcDsbA by NMR and ITC and, in parallel solved the crystal structures of TC bound to EcDsbA and VcDsbA.

### NMR studies of TC binding

**Figure 4:**
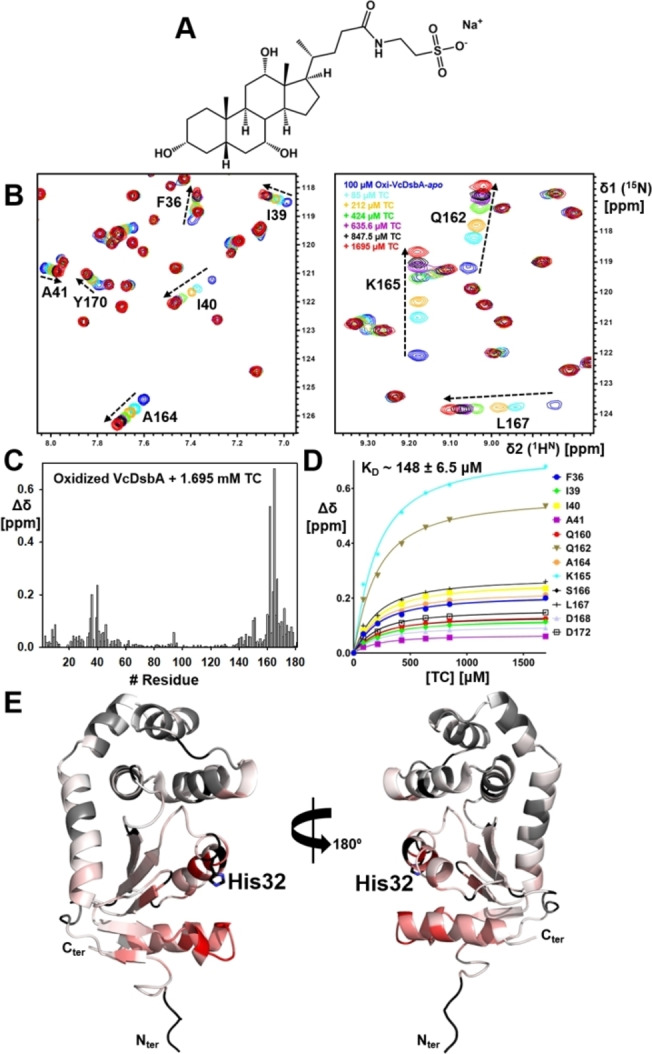
Chemical shift perturbations (CSP) observed in the ^1^H‐^15^N HSQC spectrum of oxidized VcDsbA upon addition of sodium taurocholate (TC). **A**. Chemical structure of sodium taurocholate. **B**. Chemical shift perturbations (CSP) observed in the ^1^H‐^15^N HSQC spectrum of VcDsbA with increasing concentrations of TC. TC concentrations are shown in the inset in the same colour as the corresponding spectrum. **C**. CSP histogram plot of oxidized VcDsbA (100 μM) upon addition of 1.7 mM TC. **D**. The concentration‐dependent CSP were fitted to a one‐site binding model to estimate the binding affinity (*K*_D_). **E**. CSP are mapped onto the crystal structure of VcDsbA (PDB ID: 4DVC) and are shown in colour gradient red (CSP=0.2 ppm) to white (CSP=0 ppm). Active site H32 residue is shown as sticks. Unassigned residues are shown in black.

**Figure 5:**
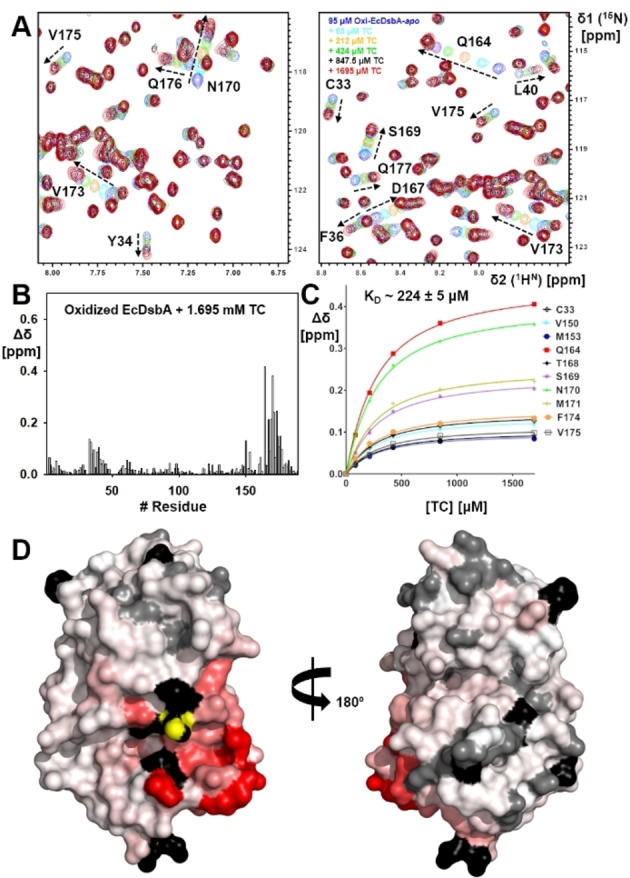
Chemical shift perturbations (CSP) observed in the ^1^H‐^15^N HSQC spectrum of oxidized EcDsbA upon addition of sodium taurocholate (TC). **A**. Chemical shift perturbations (CSP) observed in the ^1^H‐^15^N HSQC spectrum of EcDsbA with increasing concentrations of TC. TC concentrations are shown in the inset in the same colour as the corresponding spectrum. **B**. CSP histogram plot of oxidized EcDsbA (100 μM) upon addition of 1.695 mM TC. **C**. The concentration‐dependent CSP were fitted to a one‐site binding model to estimate the binding affinity (*K*_D_). **D**. CSP were mapped onto the crystal structure of EcDsbA (PDB ID: 1FVK) and are shown in colour gradient red (CSP=0.2 ppm) to white (CSP=0 ppm). Active site H32 residue is shown in yellow. Unassigned residues are shown in black.

Uniformly ^15^N‐labeled oxidized VcDsbA (100 μM) and EcDsbA (95 μM) were titrated with increasing concentrations of TC at 298 K (Figures 4 and 5). The solubility and quality of TC were confirmed by 1D ^1^H NMR and 2D ^1^H‐^1^H NOESY (Figures S5 and S6). The ^1^H‐^15^N HSQC spectra revealed that TC perturbed residues in the hydrophobic groove and the active site of VcDsbA (Figure 4B, C). TC also shifted residues in the hydrophobic groove of EcDsbA (Figure 5A, B), producing similar patterns of CSP with each enzyme (Figure 4B, C *vs*. 5 A, B). CSP were mapped onto each DsbA crystal structure as illustrated in Figures 4E and 5D. Binding affinities for TC were measured by ^1^H‐^15^N HSQC‐NMR titrations, from which *K*_D_ values were determined to be 148±6.5 μM for VcDsbA and 224±5 μM for EcDsbA (Figure 4D and Figure 5C). ITC measurements yielded *K*_D_ values of 112±11 μM and 428±44 μM for VcDsbA and EcDsbA, respectively (Figure S7). The discrepancy between the NMR and ITC‐derived values is not unexpected for low affinity interactions such as these. The larger differences between these values and those reported by Xue *et al*. may reflect the different solution conditions used in the respective studies.

These data confirm that TC binds to the hydrophobic groove of both DsbA enzymes, and with slightly higher affinity in the case of VcDsbA. Although it has been suggested that high concentrations of bile salts may cause protein unfolding,^[24]^ there is no evidence of this in the HSQC spectra for either protein even at the highest TC concentration tested (∼1.7 mM). In both cases resonances show linear CSP upon titration with TC, and peaks with CSP >0.05 ppm correspond to residues around the hydrophobic groove, suggesting two‐state exchange consistent with 1 : 1 binding. The NMR data also revealed that, under the conditions used for the titrations, the TC did not induce protein unfolding and had no effect on the oxidation state of either DsbA, as there was no indication of resonances characteristic of the reduced form of either protein.

### Crystal structures of TC in complex with EcDsbA and VcDsbA

**Figure 6:**
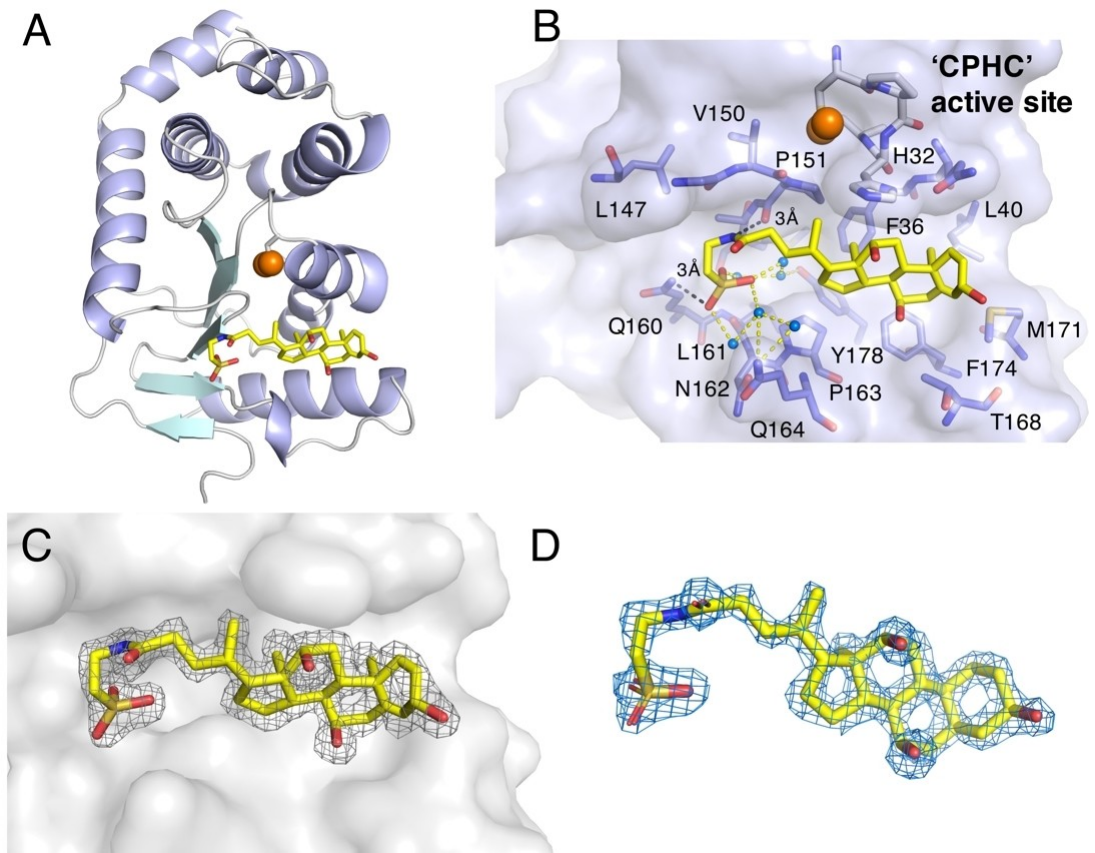
Crystal structure of oxidized EcDsbA in complex with sodium taurocholate (TC). **A**. Cartoon model of the EcDsbA‐taurocholate complex. Sulfur atoms of redox active Cys30 and Cys33 are shown as orange spheres. α‐helices are shown in light purple and β‐strands are shown in cyan. **B**. A cluster of residues within 5 Å of taurocholate are shown as sticks and labelled. Sulfur atoms of redox active Cys30 and Cys33 are shown as orange spheres. CPHC active site is indicated in the structure. The distance of polar interactions is labelled and shown as black dashed lines. Water‐mediated hydrogen bond network is shown as yellow dashed lines. **C**. 2F_o_‐F_c_ electron density map contoured at 1σ is shown as grey mesh. EcDsbA is shown as grey surface. **D**. Simulated annealing omit σ_A_‐weighted mF_o_‐DF_c_ electron density map contoured at 3.0σ is shown as blue mesh.

**Figure 7:**
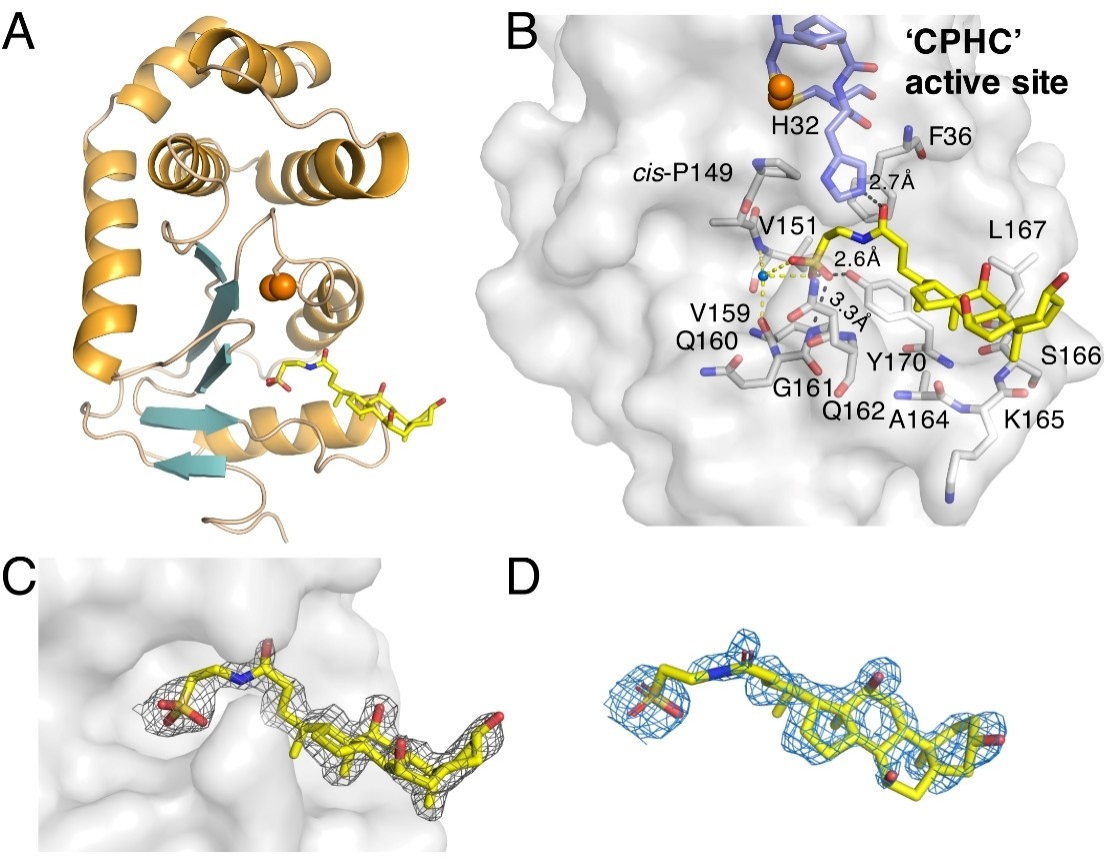
Crystal structure of oxidized VcDsbA in complex with sodium taurocholate (TC). **A**. Cartoon model of the VcDsbA‐taurocholate complex. Sulfur atoms of redox active Cys30 and Cys33 are shown as orange spheres. α‐helices are shown in orange and β‐sheets are shown in cyan. **B**. A cluster of residues within 5 Å of taurocholate is shown as sticks and labelled. The distance of polar interactions is shown as black dashed lines. Water‐mediated polar interactions are indicated by yellow dashed lines. **C**. 2F_o_‐F_c_ electron density map contoured at 0.7σ is shown as grey mesh. VcDsbA is shown as grey surface. **D**. contoured at 2.0σ is shown as blue mesh.

To identify the structural details of the interaction between TC and DsbA, we solved the crystal structures of TC in complex with oxidized EcDsbA and VcDsbA using molecular replacement at resolutions of 1.79 Å and 1.74 Å, respectively (Table S1). Crystals were obtained by co‐crystallization and each asymmetric unit contained a single DsbA molecule with TC bound (Figure 6 and Figure 7) as confirmed by omit maps (Figure 6D, 7D, S10).

In the complex with EcDsbA, TC occupies the full length of the hydrophobic groove and buries 435 Å^2^ of solvent accessible surface area (SASA). It interacts with H32 in the active site and *cis*‐P151, both of which are highly conserved in DsbA enzymes and critical for disulfide catalysis (Figure 6B). The interaction with the steroid core at the right‐hand side of the groove (proximal to the active site) is driven by hydrophobic interactions with F36, L40, M171, F174, P163 and L161. On the left‐hand side (distal to the active site), the negatively charged sulfonate group of TC forms a network of polar interactions with the side chain amide of Q160 and a series of water molecules, and its amide NH forms a hydrogen bond with the backbone amide of *cis*‐P151. The binding of TC does not cause major conformational change of EcDsbA, which is also generally the case in previously reported complexes with other small molecules.[^15e^, ^16^, ^25^] The complex has an r.m.s.d. value of 0.882 Å over 167 C^α^ positions compared to *apo* EcDsbA (PDB ID 1FVK) (Figure S8). Additional polar contacts are made to a symmetry related EcDsbA molecule through the amide carbonyl and hydroxyl groups of TC which point away from the hydrophobic groove (Figure S9), however no hydrophobic interactions are made to the symmetry related EcDsbA molecule and the solution HSQC NMR data are consistent with the binding mode determined by X‐ray crystallography with significant CSP located in the hydrophobic groove.

TC also binds to the hydrophobic groove of VcDsbA but adopts a different binding pose compared to the complex with EcDsbA (Figure 7A). The binding contributes to a buried SASA of 401 Å^2^, which is similar to that of the EcDsbA‐TC complex. Only partial electron density of TC was observed at 1σ, nevertheless full electron density can be clearly revealed if the 2F_o_‐F_c_ map is contoured at 0.7σ in Figure 7C. Omit map is contoured at 2.0σ as shown in Figure 7D. (2F_o_‐F_c_ map at 1σ and omit map at 2.5 σ are shown in Figure S10). The binding of TC does not cause major conformational change of VcDsbA. The complex has an r.m.s.d. value of 0.33 Å over 143 C^α^ positions compared to *apo* VcDsbA (PDB ID 4DVC) (Figure S8).^[17]^ The hydrophobic groove of VcDsbA is truncated and flatter than the groove of EcDsbA as a result of a three‐residue deletion in loop L3 and a four‐residue deletion in helix H7 (Figure 1A–B). The VcDsbA‐TC complex shows that TC binds only to the right‐hand side of the VcDsbA groove where it makes hydrophobic contacts with A164, K165, S166 and L167 of loop L3 (Figure 7A, B). This results in the polar end of TC binding directly under H32 of the active site. Its amide oxygen forms a hydrogen bond with the imidazole NH of H32 and the sulfonate forms a network of hydrogen bonds to the OH of Y170 and the backbone amide NH of G161. Close inspection of the crystal structure also showed that TC makes additional hydrophobic contacts with R107 and D121 of a symmetry‐related molecule in the crystal lattice (Figure S11). The binding site of TC in the co‐structure is further supported by the solution NMR data. As illustrated in Figure 4, residues Q162, A164, K165, L167 and Y170 of VcDsbA, which are strongly perturbed upon addition of TC in the HSQC spectra, are all located within 5 Å of TC in the crystal structure.

## Discussion

Many human pathogens, including *V. cholerae*, *Shigella flexneri*, *Neisseria meningitidis*, *Salmonella enterica*, and uropathogenic *E. coli* (UPEC), rely on Dsb redox systems to deploy virulence proteins such as adhesion factors, secretion systems and toxins required for infection.[^6^, ^26^] As DsbA is not essential for bacterial viability,^[27]^ DsbA inhibitors are not expected to impair bacterial growth, which should reduce the pressure for selection of resistant strains and potentially preserve native microbiota.^[28]^ A recent study has shown that DsbA inhibitor resistance was not induced under conditions that rapidly induced resistance to antibiotics in *Salmonella enterica* serovar Typhimurium.^[29]^ Additionally, since DsbA is localized in the bacterial periplasm, access to the protein for inhibition by small molecules is expected to be less difficult than inhibition of cytoplasmic targets. These factors make DsbA a promising bacterial target for the development of antibacterial drugs.^[6]^

DsbA enzymes are widespread, although there are important differences among the DsbA systems that exist in different bacteria.^[26a]^ Based on the surface charge and architecture surrounding the active site, DsbA proteins have been categorized into different subclasses, and those from *E. coli*, *Salmonella enterica* (SeDsbA), *Klebsiella pneumoniae* (KpDsbA) and *V. cholerae* have been classified into a common subclass, Ia.^[30]^ Given their high degree of structural similarity and the broadly conserved architecture of their hydrophobic grooves, it was postulated that DsbA enzymes in this subclass may be able to be targeted by subclass Ia‐specific small molecule inhibitors.[^30^, ^31^] The hypothesis has been supported by studies showing that compounds designed to block EcDsbA could also inhibit KpDsbA and SeDsbA.[^15f^, ^31^] KpDsbA and SeDsbA share >80 % sequence identity with EcDsbA. In contrast, the sequences of EcDsbA and VcDsbA are less conserved and share only 39 % sequence identity (72/186 residues). Nonetheless, their structures are similar and can be superimposed with an r.m.s.d. of 1.3 Å over 174 C^α^ atoms.^[32]^ Although they share similar surface features, the hydrophobic groove of VcDsbA is significantly truncated compared to that of EcDsbA, which presents a smaller surface area for interaction with substrates and small molecules (Figure 1). In this work, we performed an NMR‐based fragment screen against oxidized VcDsbA and identified benzimidazole **1** as a hit. Analogue screening then gave benzimidazole **2**, which displayed a higher affinity for VcDsbA and was found to have >8‐fold selectivity for VcDsbA over EcDsbA. This suggests the development of DsbA subclass Ia‐specific inhibitors might not be readily extended to VcDsbA. To rationalize the selectivity of this compound, we attempted to generate the structures of benzimidazole **2** in complex with both VcDsbA and EcDsbA. Due to the weak binding affinity of the compound to EcDsbA, we were unable to generate a model for the EcDsbA–benzimidazole **2** complex. The model of the VcDsbA complex shows that benzimidazole **2** binds to the hydrophobic groove of VcDsbA. Comparison of the NMR structure of benzimidazole **2** bound to VcDsbA with the apo crystal structure of EcDsbA (PDB 1FVK) provides a potential explanation for the observed selectivity. An overlay of these two structures indicates that a similar mode of binding is not possible in EcDsbA as it would result in a steric clash between the benzimidazole ring and F174 and F36 side chains of EcDsbA.

**Figure 8:**
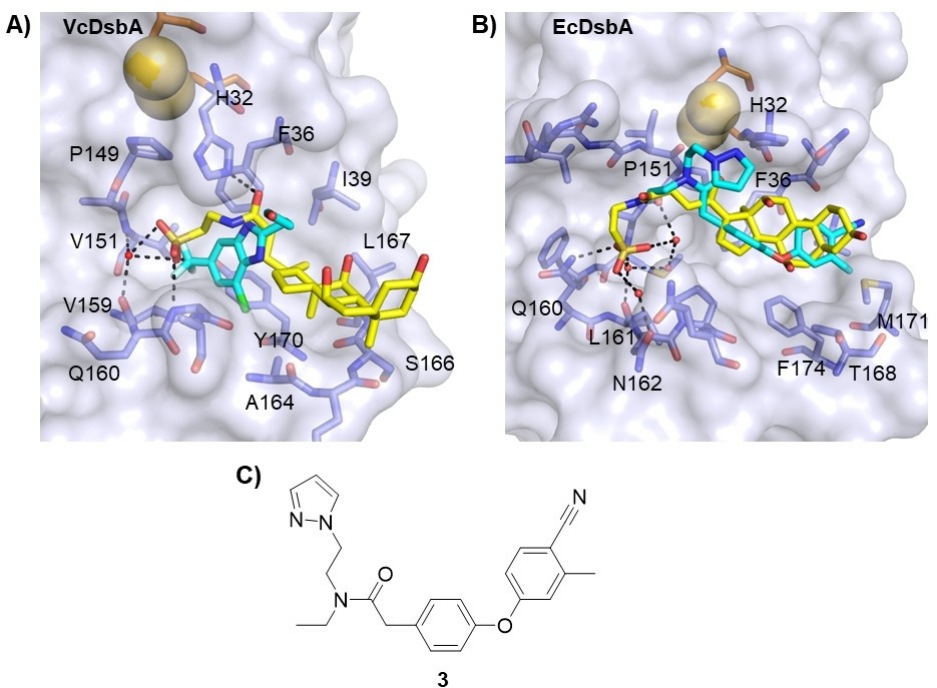
Comparison of binding poses of TC and DsbA inhibitors. **A**. Structure overlay of benzimidazole **2** (cyan sticks) with the VcDsbA (white surface, blue sticks)‐TC (yellow stick) complex. **B**. Structural overlay of the complexes of diaryl ether **3** (cyan sticks) with the EcDsbA (white surface, blue sticks)‐TC (yellow) complex (PDB ID: 6PDH). In both cases the active site Cys30 and Cys33 residues are shown as orange sticks, sulfur atoms shown as yellow spheres. Residues within 6 Å of TC are shown as blue sticks and interacting waters as red spheres with key residues labelled. Polar contacts are shown as dashed black lines. **C**. Chemical structure of diaryl ether **3**.

TC is a component of bile salts secreted in the gastrointestinal tract and has been reported to bind to and inhibit both VcDsbA and EcDsbA.^[18]^ TC binds to VcDsbA with slightly greater affinity than EcDsbA. Therefore, TC offered an opportunity to elucidate a structural basis for the binding specificity observed for these two subclass Ia DsbA enzymes. We characterized the interactions of TC with VcDsbA and EcDsbA by NMR and X‐ray crystallography and confirmed the slight selectivity for VcDsbA over EcDsbA in biophysical binding assays. The crystal structures revealed that TC adopts different binding poses in complex with EcDsbA and VcDsbA. Comparison of these two structures showed that the 7‐residue deletion that causes truncation of the hydrophobic groove in VcDsbA significantly influences the binding pose of TC (Figure 6, 7). The side chains of F174, T168 and M171in EcDsbA are oriented towards the active site, which narrows the right‐hand side of the hydrophobic groove forming a small hydrophobic pocket around TC, pushing TC further towards the left‐hand end of the groove where additional interactions are formed. In comparison, the side chains of VcDsbA residues S166 and K165, which are at similar spatial positions to F174 and T168 in EcDsbA, are oriented away from the active site, producing a more open right‐hand side of the groove allowing the steroid core of TC to form hydrophobic contacts with the hydrophobic side chains of residues L167 and Y170. As a result, whilst the steroid core of TC interacts with residues in loop L3 of VcDsbA, the more open conformation results in the molecule binding towards the right‐hand side of the hydrophobic groove. In addition to hydrophobic interactions, the taurine portion of TC engages in several polar interactions. The VcDsbA‐TC interface consists of three direct polar interactions to the backbone amide of Gly161 and the side chains of His32 and Tyr170, whereas the EcDsbA‐TC interface has two direct polar interactions to the backbone of Pro151 and the side chain of Gln160. In addition, there are a number of water‐mediated polar interactions in each complex (Figure 8). These differences may contribute to the moderate binding selectivity of TC for VcDsbA over EcDsbA.

Comparison of the crystal structure of VcDsbA‐TC complex and the NMR model of VcDsbA‐ benzimidazole **2** complex shows the benzimidazole overlays with the taurine end of the TC directly under H32, suggesting this site could be a hot spot for interaction with VcDsbA (Figure 8A). The structural comparison of TC and benzimidazole **2** binding modes indicates expansion at the 2 or 3‐position of benzimidazole **2** with hydrophobic moieties may make additional hydrophobic contact with L3 similar to the steroid core of TC. In addition, the polar interactions that TC makes with H32 and P151 could potentially be exploited in the benzimidazole series through replacement of the trifluoromethyl and further expansion of the core (Figure 8). Comparison of the crystal structure of TC bound to EcDsbA with previously reported co‐structures of EcDsbA in complex with diaryl ether **3** ^[25]^ shows the steroid core of TC makes similar hydrophobic interactions to diaryl ether **3** in the hydrophobic groove. TC reveals opportunities for expansion of these ligands to make similar polar interaction with Q160 and structural waters that may prove useful for designing diaryl ether analogues of higher affinity. Based on the current selectivity data, it is also anticipated that DsbA inhibitors that bind with similar affinity to EcDsbA and VcDsbA will be difficult to design.

The structures of TC in complex with two DsbA proteins provide structural data to facilitate our efforts to develop higher affinity small molecule inhibitors. However, the biological relevance of the ability of DsbA to bind TC is potentially interesting in its own right. When *E. coli* or *V. cholera* enter the gastrointestinal tract, they encounter a high concentration of bile salts. Small molecules with molecular weight <700 Da, such as TC in this case, can access the periplasm via porins of the outer membrane, where they would encounter DsbA.^[33]^ Bile salts are known to be hijacked as host signals to activate *V. cholerae* virulence.^[34]^ It has been suggested that the binding of TC to VcDsbA induces virulence gene expression.[^18^, ^35^] In addition, an *E.coli* periplasmic protein UgpB has recently reported to act as a molecular chaperone by inhibiting protein aggregation induced by the bile salt cholate.^[36]^ UgpB contains a deep surface groove which potentially binds to bile salts. Based on our structures, it is possible that DsbA enzymes could also exhibit an anti‐aggregation activity by directly binding to the bile salt TC. Therefore, DsbA may have multifaceted roles in mediating bacterial virulence and fitness in addition to its classic disulfide bond formation activity.

## Conclusion

In summary, fragment hits identified through an NMR‐based screen of VcDsbA, and analogue screening resulted in identification of benzimidazole 2, which was found to bind to the hydrophobic groove of VcDsbA with affinity *K*_D_=446±10 μM. Although the binding affinity of this compound is weak, our NMR model of benzimidazole 2 in complex with VcDsbA provides a structural rationale for its observed selectivity and serves as a template for future inhibitor development. Benzimidazole 2 shows >8‐fold selectivity for VcDsbA over EcDsbA despite the overall similarity of these two proteins. To better understand the structural basis of DsbA ligand binding selectivity, we also solved co‐crystal structures of TC with both VcDsbA and EcDsbA. These DsbA : TC complex structures revealed markedly different TC binding modes, which appeared to be driven by truncation of the hydrophobic groove in VcDsbA. Comparison of these NMR and X‐ray structures uncovered the molecular basis underlying selectivity of TC and benzimidazole 2 as well as suggesting potential strategies for the development of higher affinity binders.

## Experimental Section

### Expression of unlabeled VcDsbA and EcDsbA

Unlabelled VcDsbA and EcDsbA were expressed by autoinduction as previously described.[^15b^, ^37^] Pre‐expression cultures were prepared by inoculating 10 mL Luria‐Bertani (LB) broth supplemented with 50 mg/L kanamycin from frozen glycerol stocks of *E. coli* BL21(DE3) carrying the plasmid coding for VcDsbA or EcDsbA and incubating 16 h at 37 °C with agitation at 220 rpm. ZYM‐5052 media,^[38]^ using LB in place of ZYM, supplemented with 50 mg/L kanamycin in baffled conical flasks was inoculated with 1 % v/v of pre‐culture and incubated 24–30 h at 37 °C with agitation at 170 rpm, after which the cells were harvested by centrifugation at 3200×g/4 °C for 20 min. The supernatant was discarded and the pellet was stored at −20 °C for subsequent protein extraction and purification.

### Expression of uniformly ^15^N‐labeled DsbA

^15^N‐labeled VcDsbA and EcDsbA were expressed by autoinduction.^[38]^ 20x^15^NPS, 50x5052, 1 M MgSO_4_, 100 mg/mL thiamine and 1000× trace metals were prepared beforehand. Bacterial growth and expression media were prepared immediately prior to inoculation. Pre‐expression cultures were prepared by inoculating 10 mL LB broth supplemented with 50 mg/L kanamycin from frozen glycerol stocks of *E. coli* BL21(DE3) carrying the plasmid coding for VcDsbA or EcDsbA as appropriate and incubating 16 h at 37 °C with agitation at 220 rpm. ^15^N‐5052 media^[38]^ supplemented with 50 mg/L kanamycin in baffled conical flasks was inoculated with 1 % v/v of pre‐culture and incubated 24–30 h at 37 °C with agitation at 170 rpm, after which the cells were harvested by centrifugation at 3200×g/4 °C/20 min. The supernatant was discarded and the pellet was stored at −20 °C for subsequent protein extraction and purification.

### Expression of uniformly labeled ^13^C, ^15^N DsbA

400 mL of ^15^N‐MG growth media^[38]^ supplemented with 50 mg/L kanamycin in a 2‐litre baffled conical flask was inoculated with 4 mL of appropriate pre‐expression culture and incubated at 37 °C with agitation at 170 rpm. The optical density at 600 nm of the growth culture was monitored until OD_600_ approached 3.0. The growth culture was harvested by gentle centrifugation at 1800×*g*/20 °C/15 min, the supernatant was discarded and the cell pellet was gently resuspended in the ^13^C^15^N‐MG/Kan^+^ expression media. This expression culture was transferred back to the baffled conical flask and incubated at 37 °C with agitation at 170 rpm. After 15 min for adaptation of the cells to the new media and to allow assimilation of ^13^C‐labeled glucose, 1 mM IPTG was added to induce expression and the culture was incubated a further 6 h at 37 °C with agitation at 170 rpm, after which the cells were harvested by centrifugation at 3200×g/4 °C/20 min. The supernatant was discarded and the pellet was stored at −20 °C for subsequent protein extraction and purification.

### Protein extraction and purification

Each frozen bacterial pellet was thawed, resuspended in an equal volume of osmotic shock buffer (20 mM Tris (pH 9.0), 10 mM EDTA, 50 % w/v sucrose) and maintained at 4 °C with gentle stirring for 60 min, after which the cell suspension was rapidly diluted to 10 times its volume with deionized H_2_O at 4 °C. The suspension was maintained at 4 °C and stirred for a further 90 min before the cell pellet and lysate were separated by centrifugation at 50000×*g*/4 °C/30 min. The lysate was carefully decanted from the pellet and stored at −20 °C. The cell pellet was resuspended in 10 times its volume of lysis buffer (20 mM Tris (pH 8.0), 25 mM NaCl, 4 mg/mL colistin sulfate) and maintained at room temperature with gentle stirring for 18–24 h. This suspension was then centrifuged at 50000×*g*/4 °C/30 min and the lysate was decanted and added to the thawed osmotic shock lysate. The pellet was discarded and the target protein was purified from the combined lysate. (NH_4_)_2_SO_4_ was added to the combined lysate with gentle stirring to a concentration of 0.8 M and the solution was syringe filtered (0.22 μm, Millipore) for loading onto a HiLoad 1610 Phenyl Sepharose HP hydrophobic interaction chromatography column (GE Healthcare) using 20 mM Tris (pH 8.0), 50 mM NaCl, 1 M (NH_4_)_2_SO_4_. The bound proteins were eluted with a 1–0 M (NH_4_)_2_SO_4_ gradient. Fractions were analyzed by SDS‐PAGE and those containing target protein were pooled and concentrated to 10 mL using an Amicon centrifugal diafiltration unit (3000 MWCO, Millipore) and buffer exchanged to 50 mM HEPES, pH 6.8, 50 mM NaCl using a HiPrep 2610 desalting column (GE Healthcare). Following buffer exchange, the fractions containing target protein were pooled and loaded onto a MonoQ HR 10/10 anion‐exchange column (GE Healthcare). In these buffer conditions, the purified target protein was collected in the flowthrough fraction and impurities were eluted using a 50 mM–1 M NaCl gradient. Protein purity was confirmed by SDS‐PAGE.

### Oxidation of DsbA

Following purification by anion‐exchange chromatography, the target protein solution was concentrated by diafiltration (Amicon 3000 MWCO, Millipore) and treated with 10‐molar excess of freshly prepared 15 mM copper‐phenanthroline solution. After 1 h reaction time at 4 °C, the copper‐phenanthroline was removed by buffer exchange using a HiPrep 2610 desalting column with Buffer A (50 mM HEPES, pH 6.8, 50 mM NaCl).

### Adjustment to final protein concentration

For NMR experiments, protein concentration was adjusted to 0.1 mM by concentration using Amicon centrifugal diafiltration units (3000 MWCO, Millipore) or dilution using NMR buffer (50 mM HEPES, pH 6.8, 50 mM NaCl). For CSP measurements using 1D ^1^H and ^1^H‐^15^N‐HSQC NMR, D_2_O was added to a final concentration of 10 % (v/v). For NOESY spectra of VcDsbA in the presence of benzimidazole 2, the protein sample was exchanged into NMR buffer (50 mM HEPES, pH 6.8, 50 mM NaCl) containing 99 % D_2_O. Final protein concentrations were quantified using a NanoVue UV spectrophotometer (GE Healthcare) based on an extinction coefficient for oxidized EcDsbA of ϵ=28545 M^−1^ cm^−1^, and for oxidized VcDsbA of ϵ=10500 M^−1^ cm^−1^.

### Sequence specific resonance assignments of VcDsbA and EcDsbA

Backbone and side chain assignments of oxidized VcDsbA have been reported previously at 320 K (BMRB under accession number 7360) and 300 K.[^37^, ^39^] We further collected triple‐resonance experiments (CBCAcoNH, HNCACB, HNCA, HNCO and HNCACO) at 298 K on a Bruker 600 MHz NMR spectrometer equipped with CryoProbe to establish sequential assignments for VcDsbA through C^α^, C^β^ and C shift correlation to backbone amides. 300 μL of 0.3 mM [U‐^13^C^15^N]‐labelled VcDsbA (50 mM HEPES, pH 6.8, 50 mM NaCl, 2 mM EDTA) in a 5 mm Shigemi tube was used for data acquisition. Data were processed using Topspin 3.2 (pl 7) and analyzed using CARA.^[40]^ Stereospecific assignment information for valine and leucine sidechain methyl groups was previously available^[37]^ and they were used for structure determination of VcDsbA‐benzimidazole 2 complex using HADDOCK. We previously reported the backbone assignments of oxidized EcDsbA,^[15b]^ and they were used for the chemical shift perturbation analysis in 2D ^1^H‐^15^N HSQC spectra.

### Fragment Screening of oxidized VcDsbA by STD NMR Spectroscopy

For ligand‐based screening by STD NMR, unlabelled oxidized VcDsbA was prepared at 10 μM in 10 mM HEPES (pH 7.0), 150 mM NaCl buffer containing 10 % D_2_O. A 550 μL aliquot of this protein solution was added to each of one hundred screw‐top Eppendorf tubes, each containing a cocktail comprising 1 μL each of five Maybridge Library fragments in D_6_‐DMSO. The final concentration of each fragment in the cocktail was 300 μM. STD spectra were acquired at 283 K on a Bruker Avance 800 MHz spectrometer fitted with a triple‐resonance 5 mm TCI Cryoprobe equipped with single‐axis gradients and sample changer. Saturation was achieved by selective irradiation for a period of 3 s via a train of 50 ms Gaussian pulses centered at a frequency of −1 ppm. For the reference spectra a similar saturation pulse was applied 20000 Hz off‐resonance. A 20 ms spin‐lock period before acquisition allowed the protein signal to decay. The FID datasets were processed and the results were analyzed using Topspin by comparison of the STD spectra with reference 1D spectra of the individual compounds at 283 K. The fragments that gave STDs in the cocktails were identified through alignment with their reference 1D spectra.

### Hit validation and binding site identification using 2D ^1^H‐^15^N HSQC NMR

Hits from the 1D ^1^H‐STD NMR screen were validated, and the location of the fragment binding site was identified using 2D ^1^H‐^15^N HSQC NMR. A reference ^1^H‐^15^N HSQC of oxidized VcDsbA (100 μM [U‐^15^N]‐labelled protein, 2 % d_6_‐DMSO, 50 mM HEPES (pH 6.8), 50 mM NaCl) was acquired and compared with a spectrum acquired under the same conditions for a sample containing the oxidized VcDsbA in the presence of a test fragment (1 mM). In parallel, the effect of DMSO and pH on the chemical shifts of resonances in a sample of *apo*‐VcDsbA were analysed to allow for identification of chemical shift perturbations caused by fragment binding. 3 mm thick‐walled tubes of sample volume ∼160 μL were used for NMR data collection. All data were acquired at 298 K on a Bruker 600 MHz spectrometer equipped with auto‐sampler and CryoProbe. Spectra were processed by Topspin, and analyzed either by CARA^[40]^ or SPARKY.^[41]^ Weighted CSP were calculated using equation 1, ^[42]^

(1) $$CSP\left(\Delta \delta \right)=\sqrt{{{\Delta \delta }_{HN}^{2}+\left(0.2\times {\Delta \delta }_{N}\right)}^{2}}$$

where ${\Delta \delta }_{HN}$ and ${\Delta \delta }_{N}\;$ are the measured chemical shift difference of the backbone amide proton and nitrogen between *apo‐* and *holo*‐HSQC.

Affinity estimation from ligand‐detected ^19^F NMR. The change in the resonance frequency of the single ^19^F signal in the 1D ^19^F NMR spectrum of benzimidazole 2 was used to estimate the binding affinity using equation 2.^[43]^

(2) $$K_{D}=\frac{{{\gamma \left[L\right]}_{1}-\left[L\right]}_{2}}{1-\gamma }$$

where γ is described as ΔδF1/ΔδF2 and ΔδF_1_ and ΔδF_2_ denote the changes in chemical shift of the fluorine signal of benzimidazole 2 in the absence of VcDsbA and in presence of VcDsbA (10.0 μM) at ligand concentrations of [L]_1_ and [L]_2_, respectively.

### Analysis of VcDsbA – benzimidazole 2 complex using HADDOCK

The model structure of the VcDsbA‐benzimidazole 2 complex was determined using the previously described approach.^[21]^ In brief, the PDB file (PDB ID: 4DVC) for VcDsbA was optimized using PDB_REDO^[44]^ and all water molecules, incidental ions and small molecules associated with crystallization were removed. The chain ID was removed and TER and END lines were added to each PDB file. Due to the dynamic nature of the hydrophobic pocket/groove in VcDsbA, an ensemble of protein conformers was generated from the crystal structure using a protocol described previously,^[45]^ and these were considered as the starting structures for HADDOCK calculations.[^23^, ^46^] Structure files for the fragments were prepared by 3D optimization in Maestro (Schrödinger Release 2021‐4, Schrödinger, LLC, New York, NY). A set of discrete ligand conformations was generated for benzimidazole 2 to account for ligand flexibility, using the ConfGen advanced panel in Maestro. The lowest energy conformations with similar potential energy values were retained and used for HADDOCK docking. Intermolecular distance restraints for VcDsbA–benzimidazole 2 complex were calibrated using the protocol described by Shah et al.^[46]^ and are reported in Table S3. The restraints were tabulated in CNS format as input for HADDOCK. Electrostatics was defined as “on” throughout the structure calculations. On the basis of dynamics data for VcDsbA,^[37]^ the segment “162–166” was defined as fully flexible throughout HADDOCK docking. 100 docked structures were calculated in the it0 iteration, 200 structures in the it1 iteration and then 200 structures were selected for a final flexible refinement in the explicit water iteration itw. At the end of each docking run, a family of 10 models of the complex with lowest HADDOCK score and restraint violation energy was examined manually in PyMOL.

### X‐ray data collection and structure determination

For the EcDsbA‐TC complex, TC was dissolved in 100 % methanol at a concentration of 100 mM. 10 μL of this compound solution was mixed with 90 μL of 15 mg/mL EcDsbA and incubated at 4 °C overnight. Crystals of the complex were grown at 20 °C using the hanging drop vapor diffusion method from drops containing 1 μL of the complex and 1 μL of reservoir solution (100‐200 mM KBr, 28–33 % PEG2000 MME).^[15b]^ The crystals were cryo‐protected by transferring to the reservoir solution and flashed‐cooled in liquid nitrogen. For the VcDsbA‐TC complex, TC powder (equivalent to 10 mM final concentration) was directly added to 7 mg/mL VcDsbA and incubated at 4 °C overnight. Crystals of the complex were grown at 20 °C using the hanging drop vapor diffusion method from drops containing 1.2 μL of the complex and 0.8 μL of reservoir solution (0.1 M Tris‐HCl, pH 8.5, 0.2 M MgCl_2_, 26–36 % PEG4000). The crystals were cryo‐protected by transferring to a solution of 30 % PEG4000, 0.1 M Tris‐HCl, pH 8.5, 15 % glycerol and flashed‐cooled in liquid nitrogen.

Datasets were collected on the MX2 beamline at the Australian Synchrotron using *Blue‐Ice* software. The MX2 beamline was equipped with an ADSC Quantum 315r detector. 1° oscillation images were collected for a total of 180°. All datasets were indexed and integrated with *iMOSFLM* ^*[48]*^ and were scaled using *AIMLESS*.^[49]^ Phasing was performed by molecular replacement with *Phaser MR* using a previously solved structure of *Ec*DsbA (PDB code: 1FVK) as a search model.^[50]^ The structures were completed by iterative cycles of model building and refinement using *Coot* ^[51]^ and *Phenix*.^[52]^ Data collection and refinement statistics are summarized in Table S1. Generation of molecular figures was carried out with PyMOL v1.7.2.1.

### Isothermal titration calorimetry analysis

ITC was performed using a MicroCal iTC_200_ instrument (GE Healthcare). The sample cell was loaded with 250 μL of oxidized DsbA at 100 μM in 25 mM HEPES, pH 7.4, 50 mM NaCl, 2 % DMSO. The syringe was filled with 2 mM sodium taurocholate (Sigma Aldrich) in the same buffer solution. Titrations were conducted at 25 °C using 15 consecutive injections of 2.5 μL with 180 s intervals and a stirring speed of 700 rpm. In every experiment, an initial 0.4 μL of ligand was injected to avoid slow leakage of titrant and this data point was omitted from data analysis. The association constant (*K_A_* =1/*K*_D_), enthalpy (ΔH), and entropy (ΔS) were calculated by fitting the data to a single‐site binding model using the MicroCal Origin software. Every interaction was tested in triplicate and the values given represent mean and standard error of mean (SEM) from these triplicate analyses.

### Accession numbers

Crystal structures of the EcDsbA‐TC complex and the VcDsbA‐TC complexes have been deposited into the Protein Data bank with codes 7LUI and 7LSM, respectively.

## Conflict of interest

The authors declare no conflict of interest.

1

## Supporting information

As a service to our authors and readers, this journal provides supporting information supplied by the authors. Such materials are peer reviewed and may be re‐organized for online delivery, but are not copy‐edited or typeset. Technical support issues arising from supporting information (other than missing files) should be addressed to the authors.

Supporting Information

## Data Availability

The data that support the findings of this study are available from the corresponding author upon reasonable request.
