# Supplementary material for: Selective Binding of Small Molecules to Vibrio cholerae DsbA Offers a Starting Point for the Design of Novel Antibacterials
Source: ChemMedChem. 2022 Jan 27;17(6):e202100673. doi: 10.1002/cmdc.202100673 (PMC9305425; doi:10.1002/cmdc.202100673)
Supplement: Supplementary file 1 — Supporting Information [file CMDC-17-0-s001.pdf]

ChemMedChem

Supporting Information

Selective Binding of Small Molecules to *Vibrio cholerae* DsbA Offers a Starting Point for the Design of Novel Antibacterials

Geqing Wang[+], Biswaranjan Mohanty[+], Martin L. Williams[+], Bradley C. Doak[+], Rabeb Dhouib, Makrina Totsika, Róisín M. McMahon, Gaurav Sharma, Dan Zheng, Matthew R. Bentley, Yanni Ka-Yan Chin, James Horne, David K. Chalmers, Begoña Heras,* and Martin J. Scanlon*

Table of Contents

Figure S1. 2
Figure S2. 3
Figure S3. 4
Figure S4. 5
Figure S5. 6
Figure S6. 7
Figure S7. 8
Figure S8. 9
Figure S9. 10
Figure S10. 11
Figure S11. 12
Table S1. 13
Table S2. 15
Synthesis of benzimidazole analogues 2, 18-23 16
General experiemental 16
General Procedure A 16
General Procedure B 17
References 20

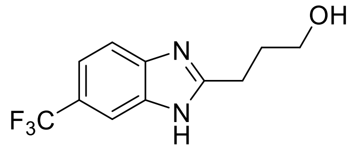

1

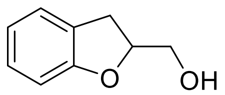

4

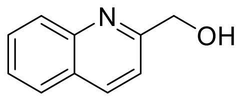

5

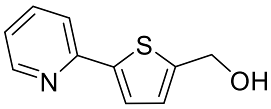

6

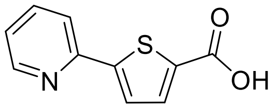

7

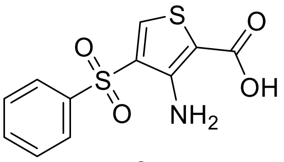

8

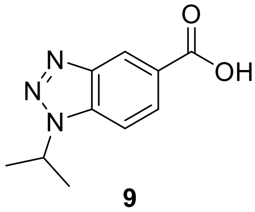

9

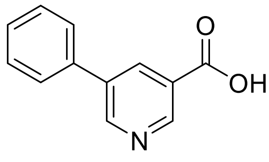

10

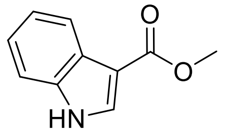

11

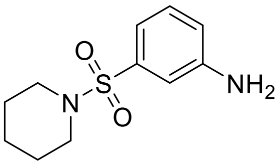

12

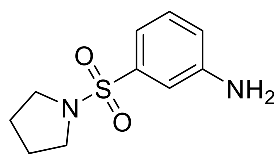

13

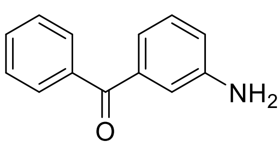

14

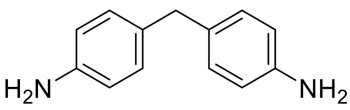

15

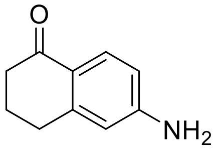

16

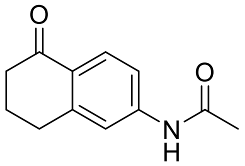

17

Figure S1. Fifteen fragment hits identified against oxidized VcDsbA by STD NMR and validated by ^{15}N-^{1}H HSQC NMR.

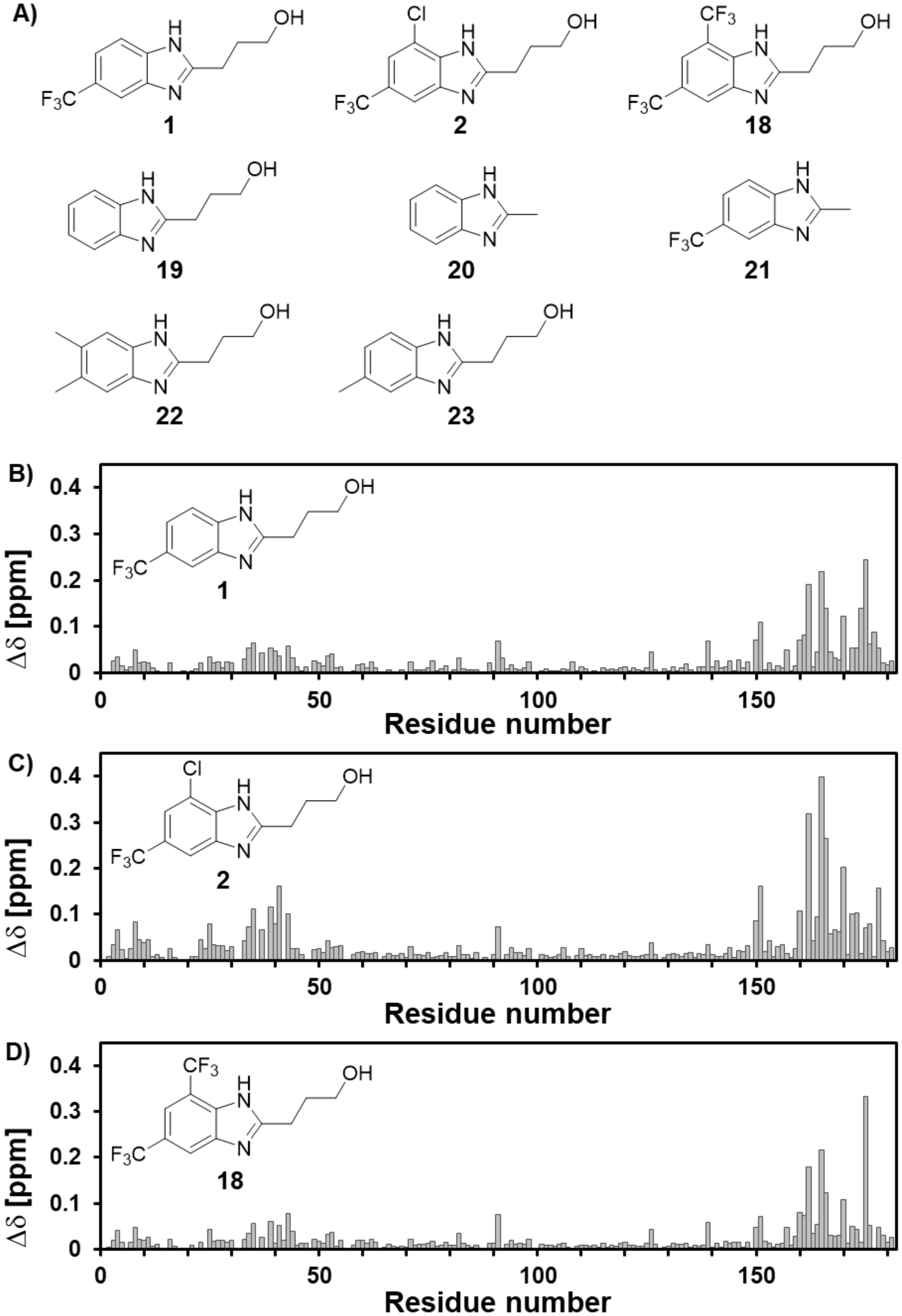

Figure S2. **Structures of Benzimidazole 1 analogues screened by ^{15}N-^{1}H HSQC NMR and CSP plots illustrating the effects on VcDsbA (100 µM) of single-point additions of benzimidazole derivatives.** B) Benzimidazole **1** (1 mM) C) Benzimidazole **2** (1 mM) D) Benzimidazole **18** (1 mM).

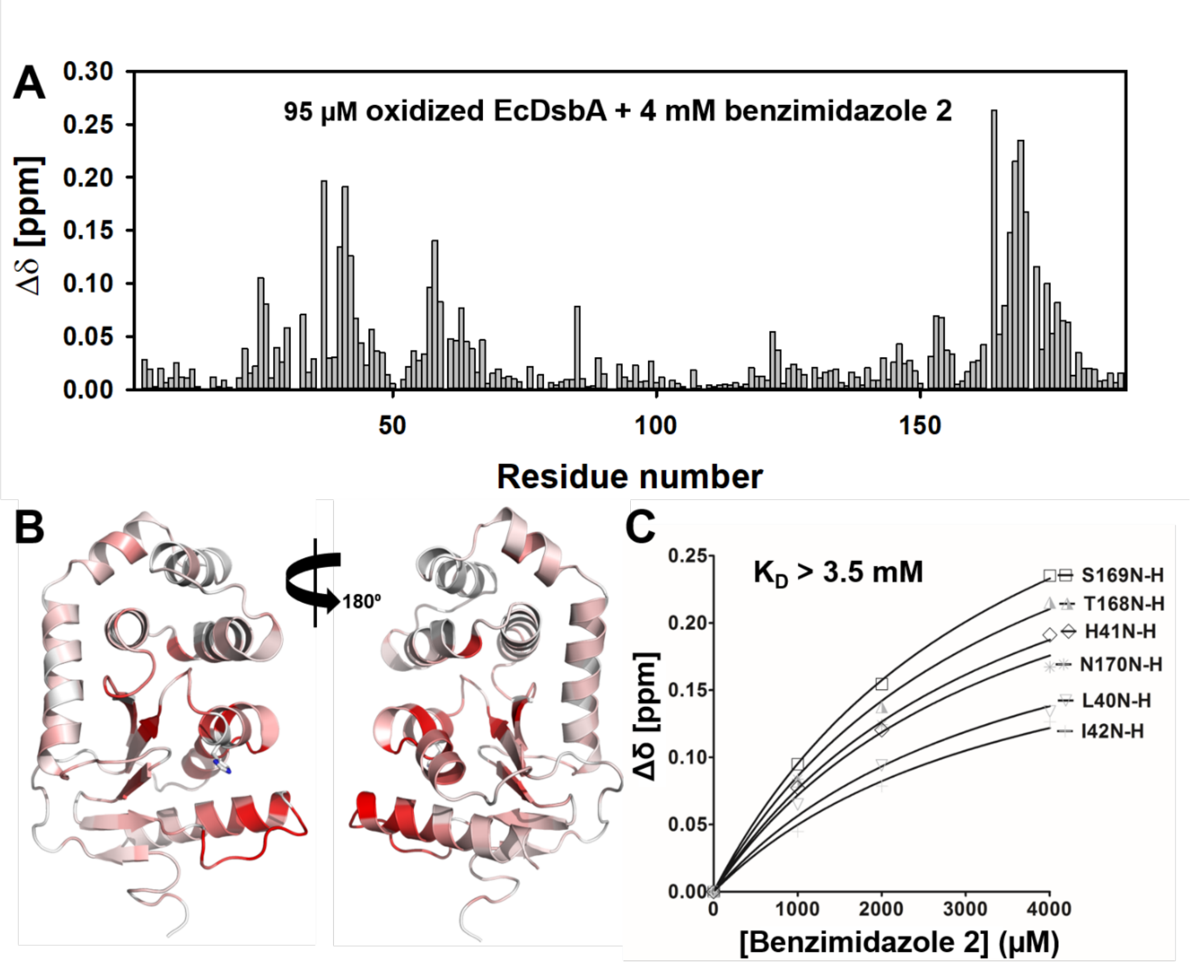

Figure S3. Chemical shift perturbations (CSP) observed in the ^{1}H-^{15}N HSQC spectrum of oxidised EcDsbA with benzimidazole 2. A. Histogram showing chemical shift perturbations (CSP) observed in the ^{1}H-^{15}N HSQC spectrum of EcDsbA (95 µM) upon addition of 4 mM benzmidazole **2**. **B.** CSP is shown as colour gradient from red (0.1 ppm) to white (0 ppm) on the crystal structure of EcDsbA (PDB ID 1FVK). Active site His32 is shown as sticks. **C.** The concentration dependence of the CSP was measured upon addition of benzimidazole **2** was used to estimate the binding affinity (K_D).

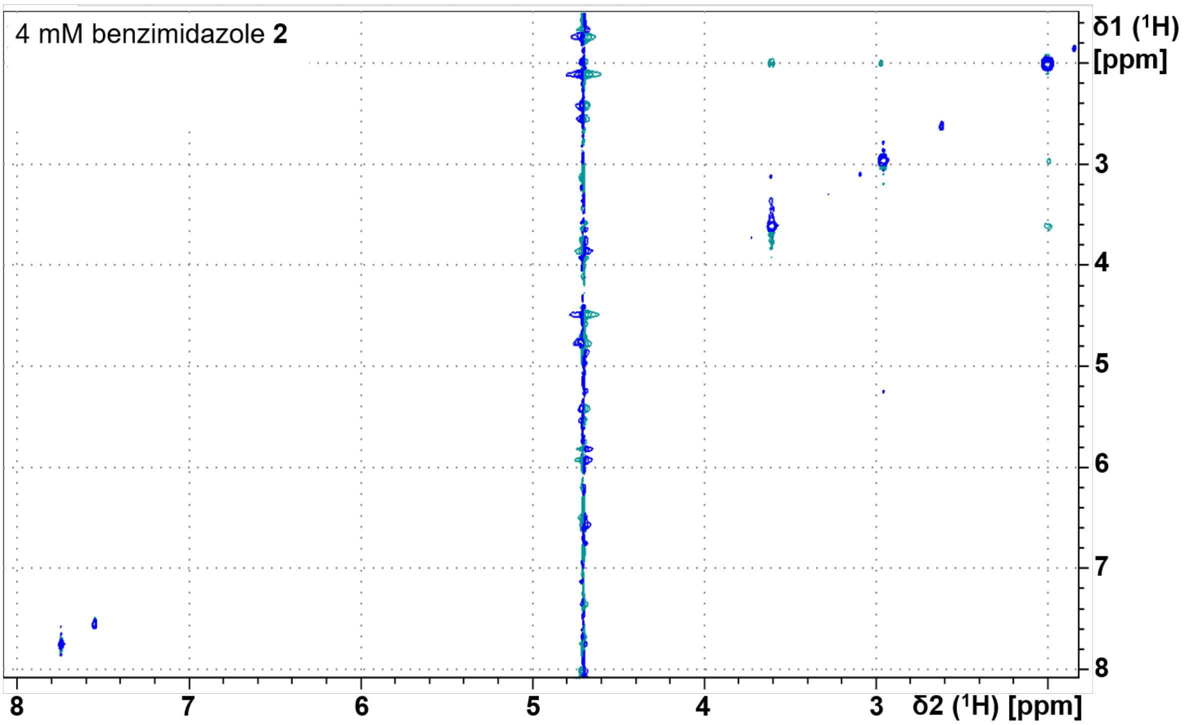

Figure S4. Solubility test of benzimidazole 2 in aqueous buffer. 2D [^{1}H,^{1}H]-NOESY of benzimidazole **2** at 4 mM in D_2O NMR buffer (50 mM NaPi, 25 mM NaCl, pH 6.5; 2% D_6-DMSO; 98% D_2O) at 298 K. NOE cross peaks are of opposite phase to the diagonal, suggesting no aggregation of benzimidazole **2** in the above solution conditions.

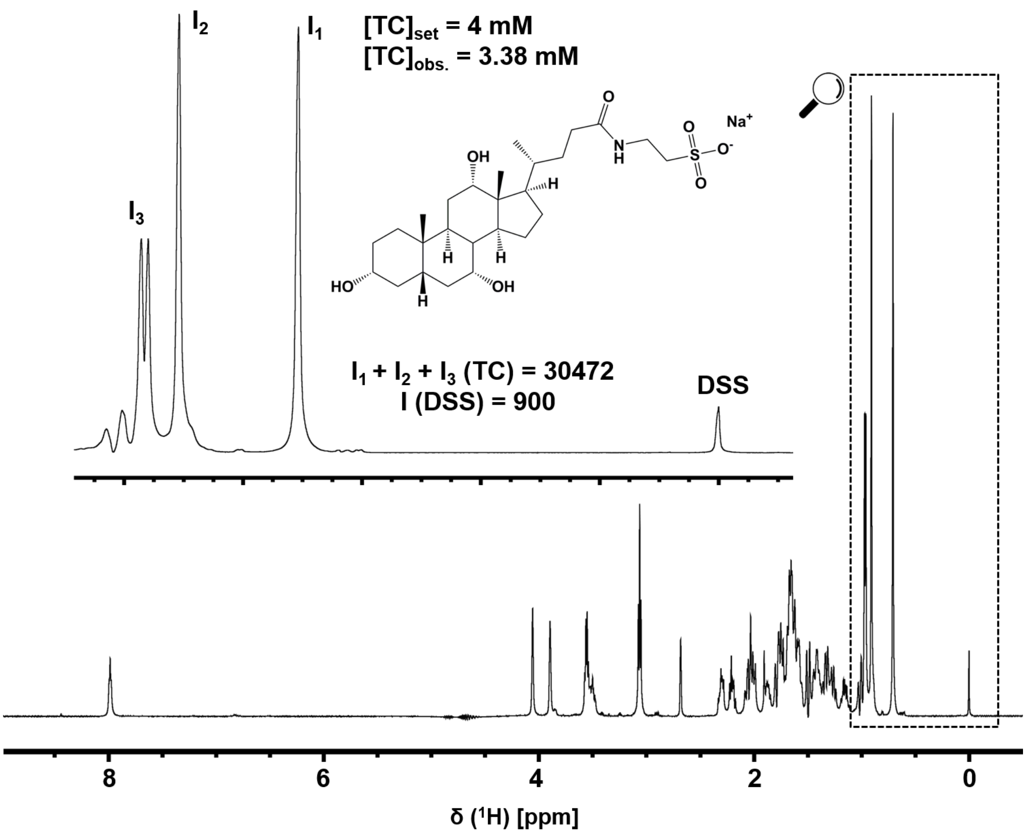

Figure S5. Quality control of bile salt sodium taurocholate (TC) by 1D ^{1}H NMR. Sodium taurocholate (TC) ($C_{26}H_{44}NNaO_7S.xH_2O$; FW 537.68; CAS 345909-26-4; T4009 (Batch#059k0110)) was obtained from Sigma-Aldrich. Since TC is hygroscopic, its stock concentration was determined by 1D ^{1}H-NMR using an internal standard of 2,2-Dimethyl-2-silapentane-5-sulfonate sodium salt (DSS), at a concentration of 100 μM prior to the interaction studies against EcDsbA and VcDsbA. TC was prepared from the stock at a notional concentration of 4 mM $[TC]_{set}$ in NMR buffer (20 mM sodium phosphate, 50 mM NaCl; 10% D_2O; 2% D_6-DMSO and 100 μM DSS) from the 200 mM stock. A 1D ^{1}H-NMR spectrum was acquired with 32K complex points and relaxation delay of 10 s at 298 K on a 600 MHz spectrometer equipped with CryoProbe, and processed by Topspin. Methyl resonances for TC and DSS were integrated by Topspin. The actual concentration $[TC]_{obs}$ was estimated at 3.38 mM from the quantitative NMR data as described previously.[1]

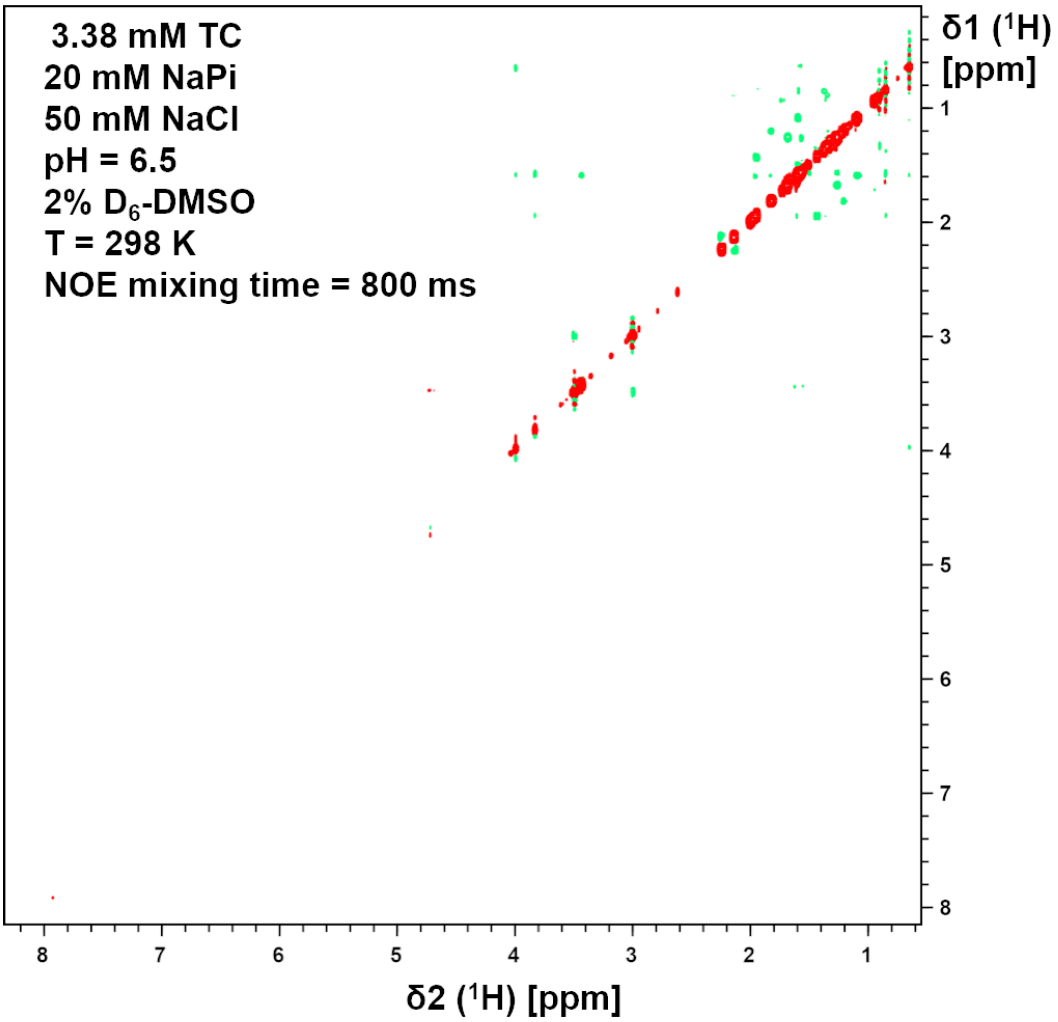

Figure S6. Solubility test of sodium taurocholate in aqueous buffer. 2D [^{1}H,^{1}H]-NOESY of taurocholate at $[TC]_{set}$ = 4 mM in D_2O NMR buffer at 298 K. NOE cross peaks are of opposite phase to the diagonal, suggesting there was no aggregation in the above solution conditions.

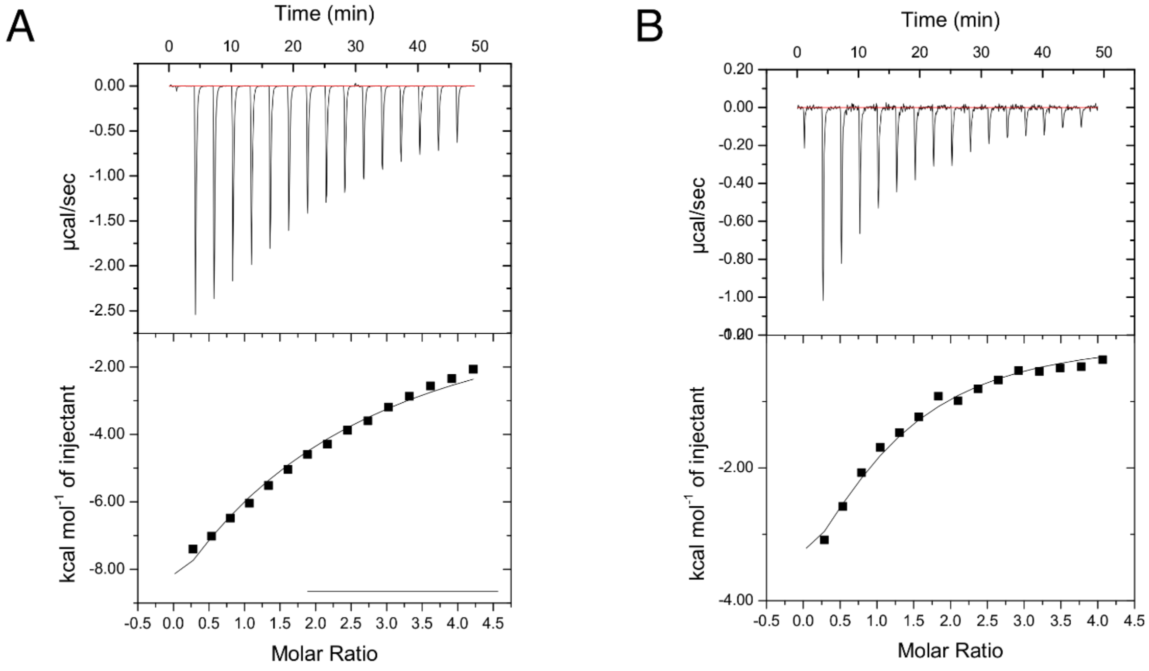

Figure S7. Binding affinity of TC against oxidized EcDsbA and VcDsbA studied by Isothermal Titration Calorimetry (ITC). ITC profile for the interactions between TC and EcDsbA (**A**)/VcDsbA (**B**). Each experiment was conducted in triplicate; a single replicate representative of the acquired ITC data is shown. For ITC data shown in panel A, N=1, K=2940 ±206 M^{-1}, ΔH= -6551 ± 273 cal/mol, ΔS=-6.11 cal/mol/deg. For ITC data shown in panel B, N=1, K=9860 ± 879 M^{-1}, ΔH= -6535 ± 240 cal/mol, ΔS=-3.64 cal/mol/deg.

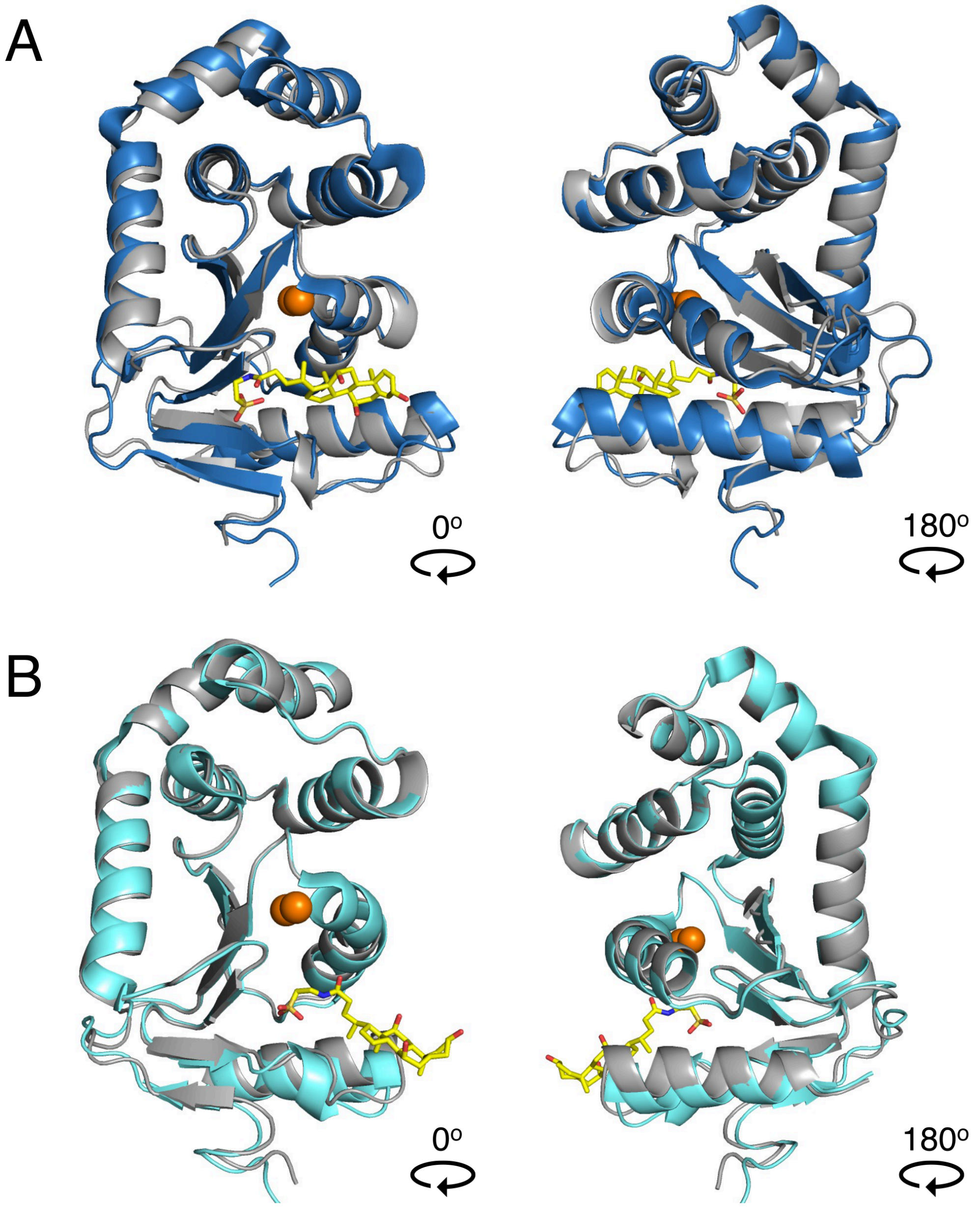

Figure S8. Overlay of crystal structures of TC-complexed DsbA and apo DsbA. A. Overlay of crystal structures of TC-complexed EcDsbA (grey) and apo EcDsbA (blue) (PDB: 1FVK). B. Overlay of crystal structures of TC-complexed VcDsbA (grey) and apo VcDsbA (cyan) (PDB ID: 4DVC).

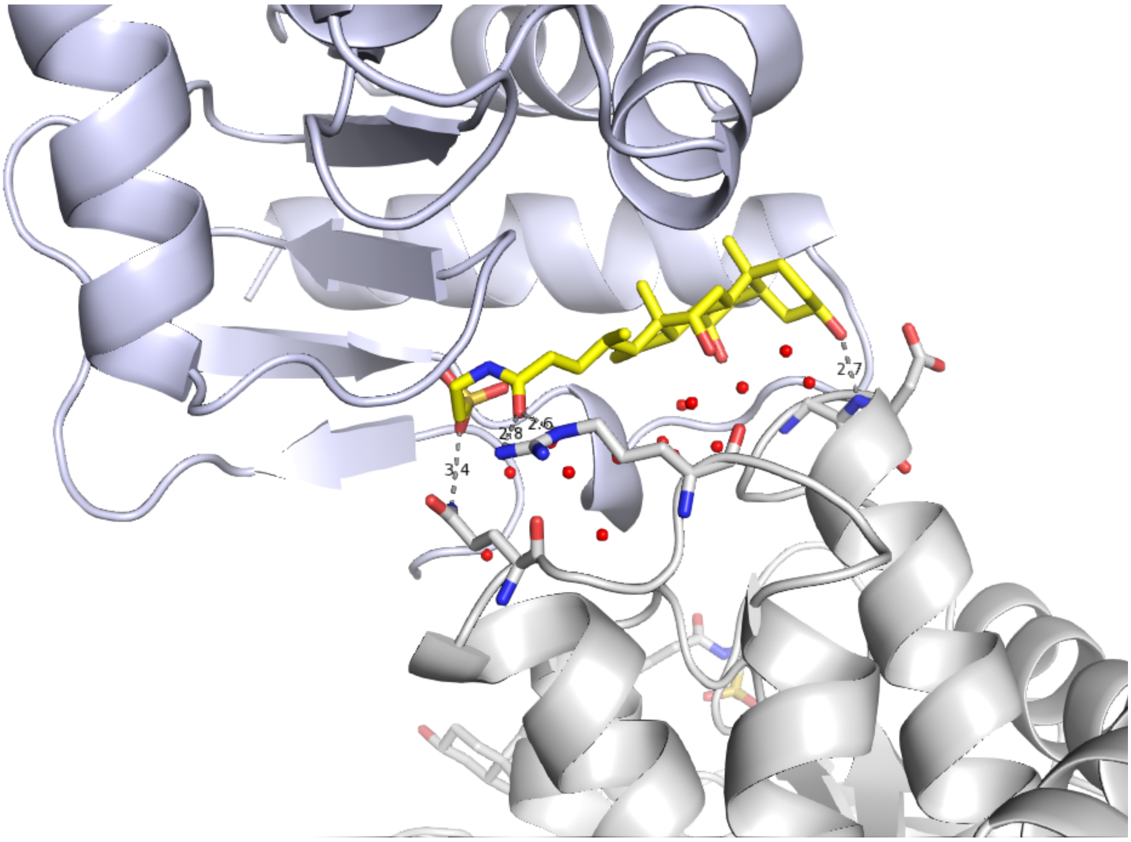

Figure S9. Crystal contacts with symmetry-related molecule in the crystal structure of the EcDsbA-TC complex. TC (yellow sticks) bound to EcDsbA (white cartoon) making additional polar contacts with a symmetry-related molecule in the crystal lattice (gray cartoon and sticks). Polar contacts are shows as dash lines in the figure with distances labelled.

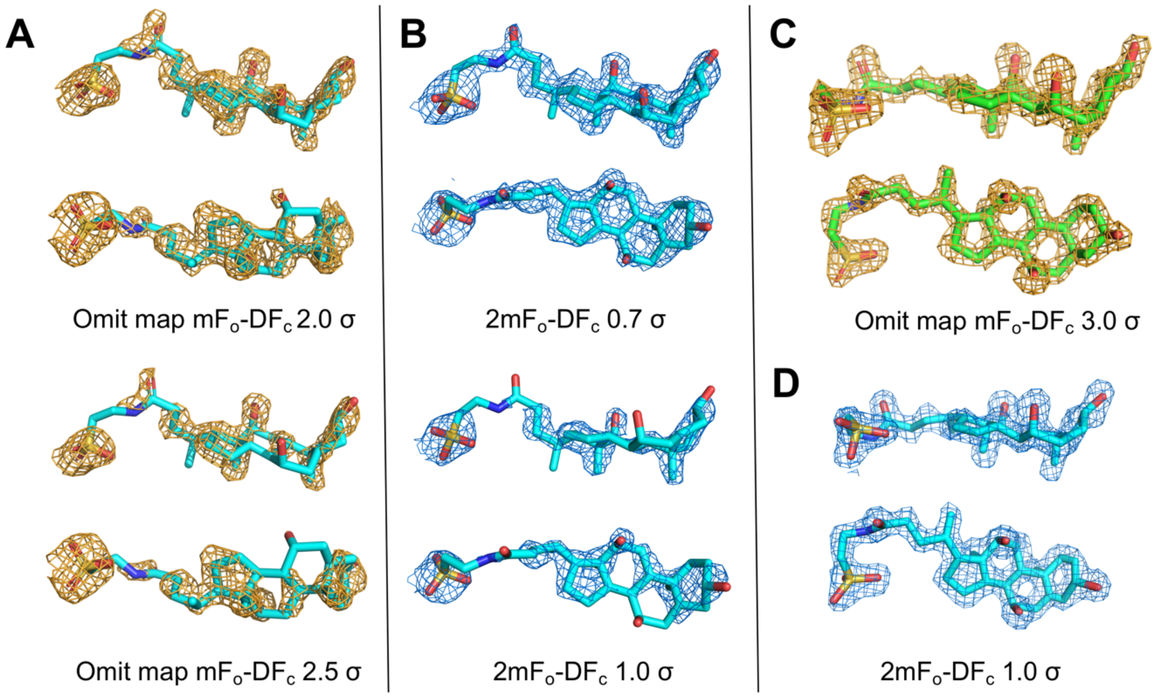

Figure S10. Electron density maps for TC in the crystal structures of VcDsbA-TC and EcDsbA-TC complexes. A. Simulated annealing omit σ_A-weighted mF_o-DF_c electron density maps for VcDsbA-bound TC contoured at 2.0σ and 2.5σ. **B.** σ_A-weighted $2mF_o-DF_c$ electron density maps for VcDsbA-bound TC are contoured at 0.7σ and 1σ. **C.** Simulated annealing omit σ_A-weighted mF_o-DF_c electron density maps for EcDsbA-bound TC contoured at 3.0σ. **D.** σ_A-weighted $2mF_o-DF_c$ electron density maps for EcDsbA-bound TC are contoured at 1σ.

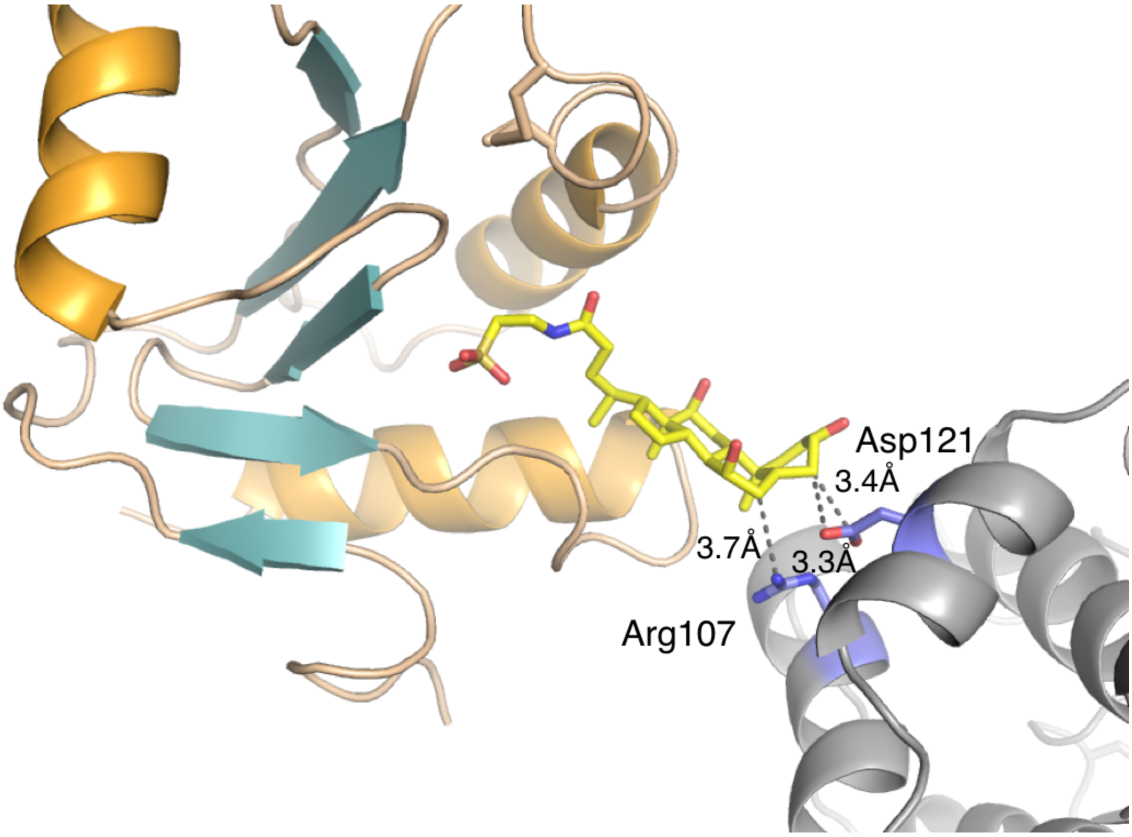

Figure S11. Crystal contacts with symmetry-related molecule in the crystal structure of the VcDsbA-TC complex. TC (yellow sticks) makes additional hydrophobic contacts with R107 and D121 of a symmetry-related molecule in the crystal lattice (gray cartoon and blue sticks). Distances between the closest atoms are shows as dashed lines in the figure with distances.

Table S1. X-ray crystallography data collection and refinement statistics.

Data collection	*Ec*DsbA-taurocholate complex (PDB: 7LSM)	*Vc*DsbA-taurocholate complex (PDB: 7LUI)
Space Group	$P2_12_12_1$	$C222_1$
Cell dimensions (Å) (a,b,c)	46.80, 50.11, 66.64	62.83, 90.34, 63.56
Angles	90.0 ,90.0 ,90.0	90.0 ,90.0 ,90.0
Resolution (Å)[a]	33.32-1.786 (1.85- 1.786)	45.17 - 1.74 (1.802 - 1.74)
Total number of observations	674660 (65246)	110027 (11217)
Number of unique observations	15426 (1521)	18866 (1844)
Multiplicity	43.7 (42.9)	5.8 (6.1)
Data Completeness (%)	99.97 (100.00)	99.68 (99.57)
$\langle I/\sigma_I \rangle$	142.47 (25.30)	17.54 (3.75)
R_{merge} [b]	0.6827 (0.8801)	0.05574 (0.3358)
Refinement		
Resolution (Å)	33.32-1.786	45.17 - 1.738
No. reflections	15424 (1521)	18865 (1844)
R_{work}[c]/ R_{free}[d]	0.1636 (0.1778) / 0.2071 (0.2549)	0.1727 (0.2044) / 0.2028 (0.2645)
No. atoms: protein	1449	1455
No. atoms: ligands	42	41
No. atoms: water	180	134
Wilson B	10.18	21.76
B factor (Å^2) – All	11.19	30.82
B factor (Å^2) – water	18.12	35.96
B factor (Å^2) – protein	10.28	29.59
B factor (Å^2) -ligands	12.87	58.17
R.m.s. deviations		
Bond lengths (Å)	0.006	0.008
Bond angles (°)	0.78	0.90
Ramachandran plot		

Residues in most favored/additionally allowed regions (%)	98.90/1.10	97.19/2.81
MolProbity Score (percentile)	1.69 (84th)	1.46 (93rd)

[a]Values in parentheses refer to the highest resolution shell.

[b]Agreement between intensities of repeated measurements of the same reflections can be defined as: $R_{merge} = \frac{\sum_{hkl}\sum_{i=1}^{n}|I_i(hkl) - \bar{I}(hkl)|}{\sum_{hkl}\sum_{i=1}^{n} I_i(hkl)}$

where $I_i\ (hkl)$ are individual values and $\bar{I}(hkl)$ is the mean value of the intensity of reflection *hkl*.

[c]$R_{fac} = \sum_h |F_o - F_c| / \sum_h |F_o|$, where F_o and F_c are the observed and calculated structure-factor amplitudes for each reflection “h”.

[d]R_{free} was calculated with 5% of the diffraction data selected randomly and excluded from refinement.

Table S2. Intermolecular NOE distance restraints used for the structure calculation of the oxidized VcDsbA—benzimidazole **2** complex.

Ligand Atom	Protein Residue	Protein Residue Atom	Target distance (Å)
H5	I39	$Q^{\delta1}$	5.02
H4	I39	$Q^{\delta1}$	5.09
H3	I39	$Q^{\delta1}$	5.46
H2	I39	$Q^{\delta1}$	4.39
H1	I39	$Q^{\delta1}$	4.45
H5	I39	$Q^{\gamma2}$	5.11
H4	I39	$Q^{\gamma2}$	4.28
H5	V159	$Q^{\gamma1}$	6.62
H4	V159	$Q^{\gamma1}$	6.04
H5	V159	$Q^{\gamma2}$	4.58
H4	V159	$Q^{\gamma2}$	5.15
H5	V151	$Q^{\gamma1}$	4.86
H4	V151	$Q^{\gamma1}$	4.97
H5	V151	$Q^{\gamma2}$	5.68
H4	V151	$Q^{\gamma2}$	5.82
H5	L167	$Q^{\delta1}$	5.28
H4	L167	$Q^{\delta1}$	4.00
H3	L167	$Q^{\delta1}$	6.07
H5	L167	$Q^{\delta2}$	5.68
H4	L167	$Q^{\delta2}$	5.13
H3	L167	$Q^{\delta2}$	6.42

Synthesis of benzimidazole analogues 2, 18-23

General experiemental

NMR spectra were recorded on a 300 MHz Bruker Avance spectrometer at 298 K. Data acquisition and processing were performed using XWINNMR (Bruker) software package version 3.5, and plotting was managed using MestRe-C software version 3.9.9.0. ^{1}H NMR spectra were referenced to an internal standard of residual proteo-solvent, δ = 3.31 ppm for methanol. Multiplicities are refere to as apparent (a), singlet (s), doublet (d), triplet (t), quartet (q), and multiplet (m). ^{13}C NMR spectra were referenced to the residual proteo-solvent, δ = 49 ppm for methanol. Overlapped ^{13}C signals were determined by ^{13}C-^{1}H HSQC and ^{13}C-^{1}H HMBC analysis and indicated as (2 C's) where appropriate. High-Resolution Mass Spectrometry was collected on a Waters Micromass LCT Premier XE time-of-flight mass spectrometer fitted with an electrospray (ESI) ion source and controlled with MassLynx software version 4.5. Low-Resolution Mass Spectrometry analyses were performed using a Micromass Platform II single quadrupole mass spectrometer equipped with atmospheric pressure (ESI/APCI) ion source. Sample management was facilitated by an Agilent 1100 series HPLC system and the instrument was controlled using MassLynx software version 3.5. Microwave reactions were conducted in a Biotage Initiator TM, in 0.5-2.0 mL vials according to manufacturer's instructions. Analytical High-Performance Liquid Chromatography (HPLC) was conducted on the Waters 2690 Separation Module coupled with a Waters 996 Photodiode Array Detector with a Phenomenex Luna 5u C8 (2) 100A (150 × 4.60 mM ID) column monitoring 254 and/or 214 nm. Buffer A: 99.9% Water, 0.1% TFA, Buffer B: 80% Methanol, 19.9% Water, 0.1% TFA. Compounds were analyzed using a Waters 2690 Separation Module by RP-HPLC using a gradient of 0-90% Buffer B in Buffer A over 20 mins followed by isocratic 90% Buffer B in Buffer A for a further 10 mins at a flow rate of 1.0 mL/min. Empower Pro software managed the processing of the samples. Thin-layer chromatography was performed on Merck Silica Gel 60 F_{254} plates. TLC plates were visualized under UV illumination at 254 nm. Column chromatography was conducted using Davisil silica gel – LC60A (40-63 microns). Ethyl acetate was distilled before use. All other solvents and chemicals were reagent grade and used as required.

General Procedure A

1,2-Phenylenediamine (1.0 eq.) and butyrolactone/carboxylic acid (1.2 eq.) were dissolved in HCl (aq. 4 M, 10 mL). The solution was heated to 100 °C for 24 hours. The reaction was then cooled to room temperature and alkalized with saturated sodium hydrogen carbonate until the solution was pH 10, followed by extraction with ethyl acetate (2 × 30 mL). The combined ethyl acetate layers were dried over anhydrous magnesium sulfate, filtered and evaporated to give

the crude product. Purification was achieved by flash chromatography in gradient mixtures of hexanes, ethyl acetate, and methanol (or as otherwise stated) to give the desired product.

General Procedure B

1,2-Phenylenediamine (1.0 eq.) and butyrolactone/carboxylic acid (1.25 eq.) were dissolved in HCl (aq. 4 M, 2.0 mL). The solution was sealed and heated to 140 °C in the microwave for 30 minutes. The reaction was then cooled to room temperature and alkalized with saturated sodium hydrogen carbonate until the solution was pH 10, followed by extraction with ethyl acetate (2 × 30 mL). The combined ethyl acetate layers were dried over anhydrous magnesium sulfate, filtered and evaporated to give the crude product. Purification was achieved by flash chromatography with gradient mixture of hexanes, ethyl acetate, and methanol (or as otherwise stated) to give the desired product.

3-(7-Chloro-5-(trifluoromethyl)-1H-benzo[d]imidazol-2-yl)propan-1-ol (2)

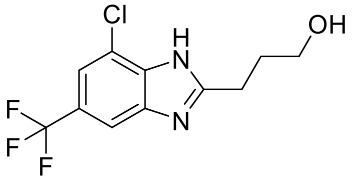

The title compound was prepared according to general procedure B with 3-Chloro-5-(trifluoromethyl)-1,2-phenylenediamine (113 mg, 0.54 mmol, 1.0 eq.) and butyrolactone (50 µL, 0.65 mmol, 1.20 eq.) to give a brown solid (28 mg, 19%). ^{1}H NMR (300 MHz CD_3OD): δ 7.74 (s, 1H), 7.50 (s, 1H), 3.66 (t, J = 6 Hz, 2H), 3.05 (t, J = 8 Hz, 2H), 2.07 (ap, J = 6 Hz, 2H). ^{13}C NMR (75 MHz CD_3OD): δ = 160.4, 139.7 (2C's), 125.5 (q, $^{1}J_{CF}$ = 270 Hz), 126.0 (q, $^{2}J_{CF}$ = 32 Hz), 121.3, 119.6, 111.5, 61.9, 31.8, 26.5. ESI-MS found 279.3, calculated 279.1 for $[M+H]^+$: TOF-ESI-HRMS found 277.0346, calculated 277.0361 for [M-H]. 98 % purity by Analytical HPLC.

3-(5,7-Bis(trifluoromethyl)-1H-benzo[d]imidazol-2-yl)propan-1-ol (18)

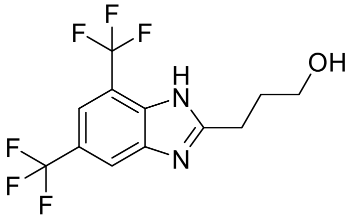

The title compound was prepared according to general procedure B with 3,5-bis(trifluoromethyl)-1,2-phenylenediamine (162 mg, 0.66 mmol, 1.0 eq.) and butyrolactone (55 µL, 0.72 mmol, 1.1 eq.) to give a dark brown solid (65 mg, 32%). ^{1}H NMR (300 MHz CD_3OD): δ 8.03 (s, 1H), 7.70 (s, 1H), 3.68 (t, J = 6 Hz, 2H), 3.08 (t, J = 8 Hz, 2H), 2.03-2.13

(m, 2H). ^{13}C NMR (75 MHz CD_3OD): δ 161.9, 141.5, 137.6, 125.3 (q, *J* = 269 Hz), 125.0 (q, *J* = 66 Hz, 2 C's), 124.8 (q, *J* = 269 Hz), 117.1 (2 C's), 62.0, 31.9, 26.6. Assignment was also validated by HSQC and HMBC confirming the overlap of peaks at 117.1 (2 carbons) and 125.3 (2 carbons). ESI-MS found 313.3, calculated 313.1 for $[M+H]^+$. 99 % purity by Analytical HPLC.

3-(1H-Benzo[d]imidazol-2-yl)propan-1-ol (19)

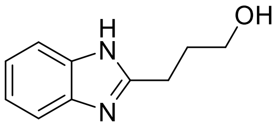

The title compound was prepared according to general procedure A with 1,2-phenylenediamine (107 mg, 0.99 mmol, 1.0 eq.) and butyrolactone (86 μL, 1.12 mmol, 1.2 eq.) to give a red solid (63 mg, 36%). Characterisation data were consistent with literature values.[2] ^{1}H NMR (300 MHz CD_3OD): δ 7.47-7.50 (m, 2H), 7.16-7.21 (m, 2H), 3.64 (t, *J*=7 Hz, 2H), 2.97 (t, *J*= 8 Hz, 2H), 2.05 (m, 2H). ESI-MS found 177.2, calculated 177.1 for $[M+H]^+$. TOF-ESI-HRMS found 177.1026, calculated 177.1022 for $[M+H]^+$. 97 % purity by Analytical HPLC.

2-Methyl-1H-benzo[d]imidazole (20)

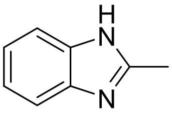

1,2-Phenylenediamine (54 mg, 0.50 mmol, 1.0 eq.) and acetic acid (28 μL, 0.50 mmol, 1.0 eq.) were heated to 110 °C for 5 hours. The reaction was then cooled and the crude mixture was recrystallized from ethyl acetate to give orange-brown crystals (17 mg, 26%). Characterisation data were consistent with literature values.[3] M.P. 176.8-178.1 °C. ^{1}H NMR (300 MHz CD_3OD): δ 7.45-7.48 (m, 2H), 7.15-7.18 (m, 2H), 2.55 (s, 3H). ^{13}C NMR (75 MHz CD_3OD): δ 152.9, 139.6, 123.1, 115.2, 14.3. ESI-MS found 133.2, calculated 133.1 for $[M+H]^+$. 99 % purity by Analytical HPLC.

3-(5,6-Dimethyl-1H-benzo[d]imidazol-2yl)propan-1-ol (21)

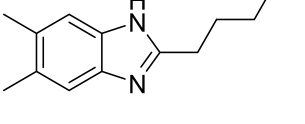

The title compound was prepared according to general procedure A with 4,5-dimethyl-1,2-phenylenediamine (104 mg, 0.76 mmol, 1 eq.) and butyrolactone (65 μL, 0.85 mmol, 1.1 eq.), to give a dark brown solid (12 mg, 8%). Characterisation data were consistent with literature values.[4] ^{1}H NMR (300 MHz CD_3OD): δ 7.25 (as, 2H), 3.63 (t, *J* = 6 Hz, 2H), 2.95 (t, *J* = 8 Hz, 2H), 2.33 (s, 6H), 1.97-2.06 (m, 2H). ^{13}C NMR (75 MHz CD_3OD): δ 155.4, 137.5, 132.4,

115.3, 62.0, 32.0, 26.2, 20.4. ESI-MS found 205.3, calculated 205.1 for $[M+H]^+$. 95 % purity by Analytical HPLC.

2-Methyl-6-(trifluoromethyl)-1H-benzo[d]imidazole (22)

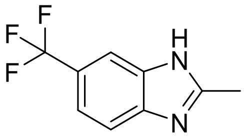

The title compound was prepared according to general procedure B with 4-trifluoromethyl-1,2-phenylenediamine (58 mg, 0.33 mmol, 1.0 eq.) and acetic acid (24 µL, 0.42 mmol, 1.25 eq.) to give an orange solid, which was purified by preparative HPLC, alkalized with NaOH (aq. 1 M, 10 mL) and extracted with ethyl acetate (3 × 10 mL). The organic combiner layers were dried over anhydrous magnesium sulfate, filtered, and evaporated to give a clear oil (10 mg, 15%). Characterisation data were consistent with literature values.[5] ^1H NMR (300 MHz CD_3OD): δ 7.78 (s, 1H), 7.61 (d, J = 8 Hz, 1H), 7.47 (dd, J = 8 Hz, 1 Hz, 1H), 2.61 (s, 3H). ESI-MS found 201.2, calculated 201.1 for $[M+H]^+$:TOF-ESI-HRMS found 201.0633, calculated 201.0634 for $[M+H]^+$. 98 % purity by Analytical HPLC.

3-(6-Methyl-1H-benzo[d]imidazol-2yl)propan-1-ol (23)

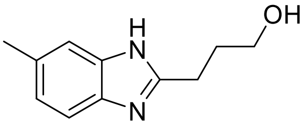

The title compound was prepared according to general procedure A with 4-methyl-1,2-phenylenediamine (110 mg, 0.90 mmol, 1.0 eq.) and butyrolactone (70 µL, 0.91 mmol, 1.0 eq.) to give a brown oil (100 mg, 58%). ^1H NMR (300 MHz CD_3OD):δ 7.37 (d, J = 8 Hz, 1H), 7.29 (s, 1H), 7.04 (d, J = 8 Hz, 1H), 3.63 (t, J = 6 Hz, 2H), 2.97 (t, J = 8 Hz, 2H), 2.42 (s, 3H), 2.03 (m, 2H). ^{13}C NMR (75 MHz CD_3OD): δ 156.0, 138.4, 136.8, 133.7, 125.2, 115.0, 114.7, 62.0, 31.9, 26.1, 21.7. ESI-MS found 191.3, calculated 191.1 for $[M+H]^+$. TOF-ESI-HRMS found 191.1184, calculated 191.1179 for $[M+H]^+$. 95 % purity by Analytical HPLC.

References

1. Taylor, A.; Doak, B. C.; Scanlon, M. J., Design of a Fragment-Screening Library. *Methods Enzymol.* **2018,** *610*, 97-115.

2. Indusegaram, S.; Katsifis, A. G.; Ridley, D. D.; Vonwiller, S. C., Nitrogen versus oxygen group protection in hydroxypropylbenzimidazoles. *Aus. J Chem.* **2003,** *56*, 819-827.

3. Infante-Castillo, R.; Rivera-Montalvo, L. A.; Hernández-Rivera, S. P., Theoritical DFT, vibrational and NMR studies of benzimidazole and alkyl derivatives. *J. Mol. Struc.* **2008,** *877*, 10-19.

4. Brown, D. W.; Mahon, M. F.; Ninan, A.; Sainsbury, M., Synthesis and oxidative behaviour of reduced indeno [1,2-b]quinoxalines and benzo[b]phenazines. *J. Chem. Soc. Perkin Trans. 1* **1995,** *1*, 3117-3124.

5. VanVliet, D. S.; Gillespie, P.; Scicinski, J. J., Rapid one-pot preparation of 2-substituted benzimidazoles from 2-nitroanilines using microwave conditions. *Tet. Let.* **2005,** *46*, 6741-6743.
